# The Landscape of Smart Biomaterial‐Based Hydrogen Therapy

**DOI:** 10.1002/advs.202401310

**Published:** 2024-08-21

**Authors:** Min Xu, Gege Wu, Qing You, Xiaoyuan Chen

**Affiliations:** ^1^ College of Biomedical Engineering Taiyuan University of Technology Taiyuan 030024 China; ^2^ Departments of Diagnostic Radiology, Surgery Chemical and Biomolecular Engineering and Biomedical Engineering Yong Loo Lin School of Medicine and College of Design and Engineering National University of Singapore Singapore 119074 Singapore; ^3^ Nanomedicine Translational Research Program NUS Center for Nanomedicine Yong Loo Lin School of Medicine National University of Singapore Singapore 117597 Singapore; ^4^ Theranostics Center of Excellence (TCE) Yong Loo Lin School of Medicine National University of Singapore 11 Biopolis Way, Helios Singapore 138667 Singapore; ^5^ Clinical Imaging Research Centre Centre for Translational Medicine Yong Loo Lin School of Medicine National University of Singapore Singapore 117599 Singapore

**Keywords:** biomaterials, hydrogen therapy, stimuli‐responsive release strategies, therapeutic mechanisms

## Abstract

Hydrogen (H_2_) therapy is an emerging, novel, and safe therapeutic modality that uses molecular hydrogen for effective treatment. However, the impact of H_2_ therapy is limited because hydrogen molecules predominantly depend on the systemic administration of H_2_ gas, which cannot accumulate at the lesion site with high concentration, thus leading to limited targeting and utilization. Biomaterials are developed to specifically deliver H_2_ and control its release. In this review, the development process, stimuli‐responsive release strategies, and potential therapeutic mechanisms of biomaterial‐based H_2_ therapy are summarized. H_2_ therapy. Specifically, the produced H_2_ from biomaterials not only can scavenge free radicals, such as reactive oxygen species (ROS) and lipid peroxidation (LPO), but also can inhibit the danger factors of initiating diseases, including pro‐inflammatory cytokines, adenosine triphosphate (ATP), and heat shock protein (HSP). In addition, the released H_2_ can further act as signal molecules to regulate key pathways for disease treatment. The current opportunities and challenges of H_2_‐based therapy are discussed, and the future research directions of biomaterial‐based H_2_ therapy for clinical applications are emphasized.

## Graphical abstract

This review provides a comprehensive overview of biomaterial‐based H_2_ therapy from the aspects of stimuli‐responsive strategies (spontaneous response, internal stimuli, and external stimuli) and therapeutic mechanisms (scavenging excess free radicals, inhibiting intracellular danger factors, and regulating signaling molecular pathways), and emphasizes the current challenges and future prospects in this field.

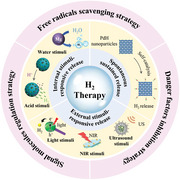

## 1 Introduction

Since the treatment of cardiovascular disease with nitric oxide (NO) was awarded the Nobel Prize in Physiology or Medicine in 1998, gas therapy has received widespread attention in the scientific community. A variety of gaseous signaling molecules such as NO,^[^ ^1^, ^2^, ^3^, ^4^ ^]^ carbon monoxide (CO),^[^ ^5^, ^6^, ^7^ ^]^ and hydrogen sulfide (H_2_S)^[^ ^8^, ^9^ ^]^ have been found to have specific biological effects and high osmotic diffusivity, which play critical roles in regulating various physiological functions, such as the nervous system, cardiovascular system, and immune system.^[^ ^10^, ^11^ ^]^ Due to the important physiological messenger effects of these gases in organisms, gas therapy is considered as a green treatment with minimal adverse toxicity to normal organs and has been extensively researched in recent years.^[^ ^12^, ^13^ ^]^ As a kind of endogenous gas, hydrogen gas (H_2_) has been traditionally known as a relatively unreactive gas. However, current studies have reported that H_2_ exhibits significant physiological/pathological regulation functions with anti‐inflammatory and anti‐tumor efficacy, which makes it a new member of therapeutic gas molecules (like NO and H_2_S).^[^ ^14^, ^15^ ^]^ More importantly, the byproduct of H_2_ in tissues is harmless water. These advantages make H_2_ therapy an emerging therapeutic strategy that can be developed for various clinical applications.

Over the past decade, H_2_ therapy has been demonstrated by more than 50 clinical trials in many oxidative stress‐/inflammation‐related diseases.^[^ ^16^ ^]^ However, its low solubility and high diffusibility preclude H_2_ gas accumulation at the lesion site, significantly affecting the further application and development of H_2_‐based therapy. Currently, the main administration routes of H_2_ include inhalation, injection of H_2_‐rich saline, drinking H_2_‐rich water, and full‐body bathing.^[^ ^17^, ^18^, ^19^, ^20^, ^21^, ^22^, ^23^, ^24^, ^25^ ^]^ The therapeutic effect of H_2_ is closely related to its concentration at the disease site. For gastrointestinal inflammation and pneumonia, inhalation of H_2_ gas and long‐term oral H_2_‐rich water can produce evident therapeutic efficacy.^[^ ^26^, ^27^, ^28^, ^29^ ^]^ However, for deep lesions, these systemic H_2_ delivery methods lack targeting, making it difficult to achieve local and sustainable high‐concentration administration, which limits the efficacy of H_2_ therapy. Thus, if the generation of H_2_ can be controlled at the disease site for sustained and powerful release, the therapeutic efficacy will be greatly improved. Fortunately, the discovery of H_2_ storage biomaterials has provided new ideas to solve these problems by efficiently encapsulating H_2_ or H_2_ prodrugs.^[^ ^30^, ^31^ ^]^ With the small size effect and easily functionalized design, these biomaterials can achieve efficient H_2_ production and responsive release in focal areas to improve the effectiveness of H_2_ therapy.^[^ ^32^, ^33^, ^34^, ^35^ ^]^ Compared with traditional drug delivery systems, biomaterial‐based stimuli‐responsive H_2_ release systems possess higher payloads and better tissue specificity, showing essential application prospects and potential clinical value.

Based on these advantages, biomaterial‐mediated H_2_ therapy has been broadly explored for various disease treatments, including cancer, inflammatory disease, cardiovascular diseases, wound healing, ischemic stroke, etc. As an emerging approach, it has significantly different therapeutic mechanisms and targets from other traditional treatments, such as surgery, chemotherapy, radiotherapy (RT), photothermal therapy (PTT), and photodynamic therapy (PDT). Thus, it is important to understand the roles of H_2_ alone and in combination with biomaterials during disease treatment. Moreover, for different diseases, the design of biomaterials and therapeutic pathways also vary greatly. Specifically, the main target of released H_2_ from biomaterials in tumor treatment is mitochondria. Owing to its bio‐reducibility, H_2_ can inhibit adenosine triphosphate (ATP) production and heat shock protein (HSP) level by impairing mitochondrial functions, thus inducing tumor cell apoptosis. For oxidative stress‐related diseases, biomaterials‐based H_2_ therapy is effective because it can restore the redox status by selectively scavenging harmful radicals and cytokines. In addition, biomaterial‐based H_2_ therapy can also act on specific intracellular pathways to treat other diseases due to its signal‐modulating capability. According to different mechanisms, H_2_‐based therapeutic strategies can be divided into three types: scavenging, inhibition, and regulation. The scavenging strategy mainly uses H_2_ to eliminate excess free radicals, such as reactive oxygen species (ROS) and lipid peroxidation (LPO), which are highly associated with oxidative damage and can destroy normal cells, eventually leading to the occurrence of various diseases. The inhibition strategy focuses on reducing the levels of intracellular danger factors, including pro‐inflammatory cytokines, ATP, and HSP. Regulation strategy is closely related to multiple signaling molecular pathways, such as lipid transport pathway, metabolic pathway, and key protein expression pathway. Generally, these mechanisms are casually associated with each other but not mutually exclusive.

**Figure 1:**
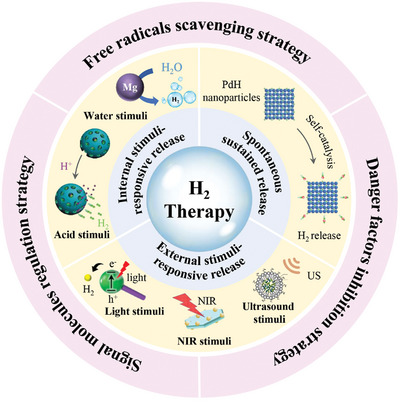
Schematic showing biomaterial‐mediated H_2_ release strategies and the therapeutic mechanism of H_2_.

Herein, this review provides a comprehensive summary of biomaterial‐based H_2_ therapy in terms of stimuli‐responsive strategies (spontaneous response, internal stimuli, and external stimuli) and therapeutic mechanisms (scavenging, inhibition, and regulation). Finally, we conclude the future directions and critical challenges of biomaterial‐mediated H_2_ therapy (**Figure** **1**).

## 2 Hydrogen Molecular Medicine

### 2.1 Development of Hydrogen Therapy

**Figure 2:**
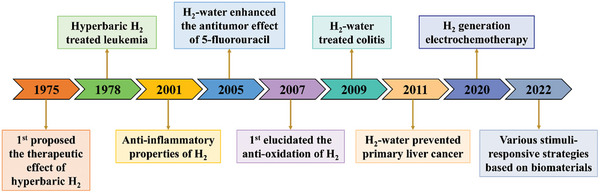
Historical development of H_2_ therapy.

For a long time, H_2_ was considered as an inert gas in the biomedical field. However, research has shown that it has biological reactivity and can effectively influence the oxidative stress state caused by diseases, thus achieving disease treatment.^[^ ^36^, ^37^, ^38^ ^]^ Dole et al. (1975) reported that hyperbaric H_2_ gas inhalation had a specific therapeutic effect on skin tumors, and the therapeutic mechanism might be related to the antioxidant effect of H_2_.^[^ ^17^ ^]^ However, in the following decades, H_2_ did not receive much attention in the biomedical field due to its flammable and explosive properties. Then, Ohsawa et al. (2007) demonstrated that H_2_ gas had a selective antioxidation mechanism, which allowed it to improve ischemia‐reperfusion injury significantly by scavenging peroxynitrite anion (ONOO^−^) and hydroxyl radicals (·OH).^[^ ^39^ ^]^ This research has raised broad interest in H_2_ therapy and driven the rapid development of research into H_2_ biology and medicine. Over the next decade, H_2_ gas has shown therapeutic effects in oxidative stress and inflammation‐associated diseases, such as cancer, cardiovascular diseases, metabolic diseases, respiratory diseases, and neurodegenerative diseases.^[^ ^40^, ^41^, ^42^, ^43^, ^44^, ^45^ ^]^ At present, H_2_ therapy, characterized by high safety and broad‐spectrum effectiveness, has become a research hotspot, and further evolved substantially “from the bench to the bedside” (**Figure** **2**).^[^ ^46^ ^]^

### 2.2 Effect of Hydrogen Therapy

Since the hypothesis that the basis of H_2_ therapy is selective neutralization of ·OH was proposed in 2007, the antioxidant action is considered to be the most prominent effect of H_2_.^[^ ^39^ ^]^ In recent years, multiple physiological effects (such as anti‐inflammatory and anti‐apoptotic effects) of H_2_ have been reported. In addition, it has been shown that H_2_ is a gaseous signaling molecule and, thus can play a signal transduction role in organisms by regulating related cellular pathways and gene expression for disease treatments.

#### 2.2.1 Anti‐Oxidant Effect

In general, oxidative stress, characterized by the excess production of ROS, especially highly cytotoxic ·OH, can damage DNA, proteins, and lipids, being closely associated with the development of diverse diseases, including ischemia‐reperfusion injury, neurodegenerative diseases, aging, cardiovascular diseases, diabetes, arthritis, and tumors.^[^ ^47^, ^48^, ^49^, ^50^, ^51^ ^]^ As a reducing gas, H_2_ scavenges highly toxic ROS, including ·OH, ONOO^−^, superoxide anion (O_2_^·−^), and hydrogen peroxide (H_2_O_2_) to effectively inhibit oxidative stress; however, it has no neutralizing effect on physiologically active free radicals. In addition to the direct neutralization reaction with free radicals, H_2_ can also act indirectly on the antioxidant systems. Recent studies have shown that H_2_ can activate endogenous antioxidant enzymes such as superoxide dismutase (SOD) and catalase (CAT) and reduce the concentrations of various oxidative stress markers such as myeloperoxidase (MPO), malondialdehyde (MDA), 8‐isoprostaglandin F2α and thiobarbituric acid with a dose‐dependent pattern in many human diseases and rodent models.^[^ ^52^, ^53^, ^54^, ^55^ ^]^

#### 2.2.2 Anti‐Inflammatory Effect

Inflammation is an organism's response to injury, including systemic and local reactions, which are complex physiological processes that aim to eliminate harmful stimuli and initiate tissue healing.^[^ ^56^, ^57^ ^]^ Studies have indicated that H_2_ can inhibit the production of inflammatory factors and increase the release of anti‐inflammatory factors.^[^ ^58^ ^]^ Nakao et al. found that breathing air containing 2% H_2_ could reduce postoperative damage and improve postoperative function after intestinal transplantation by regulating mRNA levels of interleukin (IL)−1β, IL‐6, and chemokine ligand 2 (CCL2).^[^ ^59^ ^]^ Subsequently, Ji et al. constructed a whole brain ischemia‐reperfusion rat model to prove that intraperitoneal injection of H_2_‐rich water could significantly decrease tumor necrosis factor‐α (TNF‐α), IL‐6, and nuclear factor kappa‐B (NF‐κB), and mitigate hippocampal tissue damage.^[^ ^60^ ^]^ Zhang et al. showed that H_2_‐rich saline treatment could reduce neutrophil infiltration, intercellular adhesion molecule‐1 (ICAM‐1) expression, and pro‐inflammatory cytokines levels in the local myocardial ischemia‐reperfusion rat models.^[^ ^61^ ^]^ In addition, H_2_ can down‐regulate inflammatory mediators (e.g., macrophage chemotactic protein 1) and pro‐inflammatory transcription factors (e.g., high‐mobility group box 1 (HMGB‐1) and prostaglandin E2). The anti‐inflammatory effect of H_2_ is not only reflected in the regulation of important factor levels but also can reduce the inflammatory response by increasing the activity of anti‐inflammatory cells. Recently, Chen et al. proved that H_2_ could protect mice from chronic pancreatitis by restoring the loss of regulatory T cells.^[^ ^62^ ^]^

#### 2.2.3 Anti‐Apoptotic Effect

Apoptosis is a physiological process of cell death, which is essential for the normal development and function of multicellular organisms. Abnormal control of cell death can lead to a variety of diseases, including tumors, autoimmune diseases, and degenerative diseases. Apoptosis occurs through endogenous stimulation elicited by the mitochondrial signaling pathway, or through exogenous stimulation of death receptors on the surface of the cell membrane. However, regardless of the signaling pathway, activation of the cysteinyl aspartate specific proteinase (caspase) is required, which is regarded as closely related to apoptosis. Studies have shown that inhalation of H_2_ can reduce the apoptosis number of hippocampal and cortical neuronal cells caused by cerebral ischemia‐hypoxia in neonatal rats through inhibiting caspase‐3 and caspase‐12 activities.^[^ ^63^, ^64^ ^]^ Besides, H_2_ also can reduce apoptosis by increasing the expression of anti‐apoptotic proteins, such as B‐cell lymphoma (Bcl)−2 and Bcl‐xL, and decreasing the levels of the pro‐apoptotic protein Bax.^[^ ^65^, ^66^ ^]^ Furthermore, Wang et al. found that H_2_ exhibited therapeutic effect by inhibiting pro‐apoptotic molecules such as C‐Jun N‐terminal kinase (JNK) and NF‐κB in animal models of beta‐amyloid induced Alzheimer's disease (AD).^[^ ^67^ ^]^

#### 2.2.4 Signal Transduction Effect

In recent years, several studies have shown that H_2_ as a gaseous molecule, similar to H_2_S, CO, and NO signaling molecules, plays a role in the regulation of relevant cellular pathways, including the nuclear factor erythroid 2‐related factor 2 (Nrf2)/heme oxygenase 1 (HO‐1) pathway,^[^ ^68^, ^69^ ^]^ the NF‐κB pathway,^[^ ^70^, ^71^ ^]^ the phosphatidylinositol‐4, 5‐bisphosphate 3‐kinase (PI3K)/protein kinase B (Akt) pathway,^[^ ^72^, ^73^ ^]^ and the Rho pathway.^[^ ^74^, ^75^ ^]^ Meanwhile, the effects of H_2_ on non‐coding RNAs (microRNA, miRNA) have gradually attracted the attention of researchers. It was reported that H_2_ could down‐regulate the expression of miRNA‐21, miRNA‐9, miRNA‐200, and miRNA‐210 while up‐regulating the expression of miRNA‐199.^[^ ^76^, ^77^, ^78^ ^]^ In addition, H_2_ can modulate the phosphorylation of signal transduction molecules and other protein modifications.^[^ ^79^, ^80^ ^]^ Increasing number of basic studies have investigated the therapeutic mechanisms and targets of H_2_ in tissues, cells and molecules, which provide a reference for clinical application.

### 2.3 Advantages of Hydrogen Therapy

Safety is the outstanding advantage of H_2_ for medical applications, mainly based on the following four aspects: 1) H_2_ is a type of endogenous gas primarily produced and utilized by intestinal flora. During normal cellular metabolism, H_2_ is not involved in biochemical reactions because its reducibility is weaker than the other reducing agents in cells^[^ ^81^ ^]^; 2) At present, the USA Food and Drug Administration (FDA) and China FDA have both added H_2_ to the list of permitted food additives. H_2_‐rich water has become a popular commercial functional drink worldwide, especially in Japan, China, and Korea; 3) As a breathing gas, H_2_ has been used in diving medicine.^[^ ^82^ ^]^ The world's deepest dive record was made by breathing an H_2_‐O_2_ mixture, which is significantly safer than oxygen; 4) H_2_ gas reacts with other substances to produce water, while the excess H_2_ can be excreted by breathing without any residue. To date, no toxic side effects of H_2_ have been reported.

Unlike most traditional chemical drugs, H_2_ has high diffusivity across biological membranes because of its small size and lack of polarity, allowing it to reach internal cell structures, including mitochondria and nuclei.^[^ ^83^ ^]^ Besides, the high diffusivity makes H_2_ cross the blood‐brain barrier (BBB) easily to treat central nervous system (CNS) diseases.^[^ ^84^ ^]^ Compared with other antioxidants, H_2_ has a higher tolerance and does not affect the physiological parameters of the blood (such as temperature, blood pressure, and blood oxygen). Taken together, all these properties of H_2_ make it a promising strategy for treating various diseases.

## 3 Controlled Release of Hydrogen Based on Biomaterials

The H_2_ therapeutic efficacy is highly correlated with the gas concentration at the disease site. Due to its low solubility and strong volatility, H_2_ is difficult to accumulate at high concentrations in focal tissues for long periods. At present, there are three main means for H_2_ therapy: direct breathing of low‐concentration H_2_, drinking saturated H_2_ water, or injecting saturated H_2_‐containing saline. Recently, some emerging methods of H_2_ therapy such as topical drip or H_2_ bath and inducing colon bacteria to produce H_2_ have also been gradually developed. However, the existing approaches are not able to control the sustained and effective release of H_2_ at the lesion sites. In the past decades, the rapid development of responsive biomaterials has provided essential platforms for achieving targeted and efficient H_2_ release to diseased tissues, thereby improving the therapeutic efficacy of H_2_. The H_2_ released by biomaterials comes from a variety of sources, some of which are directly adsorbed H_2_ gas or hydrogen atoms formed after H_2_ adsorption. Some released H_2_ derives from the reaction of biomaterials with endogenous substances, such as hydrogen ions (H^+^) in acidic conditions and water in the environment. In addition, other biomaterials can generate H_2_ from protons provided by some substances, such as L‐ascorbic acid (AA) and glutathione (GSH). Specifically, protons from these substances can accept electrons produced by biomaterials in response to exogenous stimuli, thereby releasing H_2_. Importantly, the released H_2_ from biomaterials exists in the form of gas and remains the gas diffusion effect in lesion sites, avoiding the shortcoming of limited effective area for traditional therapeutic modalities. Therefore, responsive biomaterials provide conditions for the controlled release and targeted transport of H_2_, which is scientifically important for realizing efficient and low‐toxicity disease treatment.

**Figure 3:**
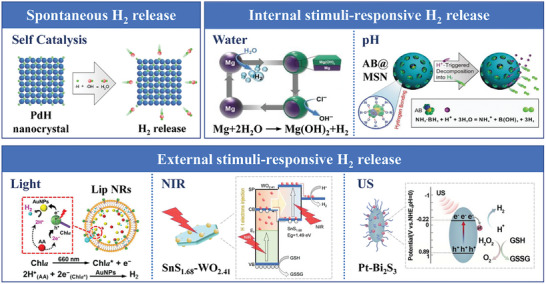
Overview of typical biomaterial‐based H_2_‐controlled release strategies. Biomaterials constructed with stimuli‐responsiveness can release H_2_ through spontaneous responses, internal stimuli, and external stimuli.^[^ ^101^, ^107^, ^110^, ^115^, ^126^, ^136^ ^]^ Copyright 2017, American Chemical Society. Copyright 2018, Nature Publishing Group. Copyright 2021, Nature Publishing Group. Copyright 2022, Wiley‐VCH. Copyright 2018, Elsevier. Copyright 2018, Wiley‐VCH.

In this section, we summarized the biomaterial‐based controlled H_2_ release strategies from three aspects, including spontaneous release, internal stimuli‐responsive release, and external stimuli‐responsive release (**Figure** **3**). The typical biomaterials used for H_2_ therapy are summarized in **Table** **1**.

**Table 1:** Summary of biomaterials‐medicated stimuli‐responsive strategies for hydrogen therapy.

Stimuli‐responsive strategies		Biomaterials	Source of H_2_	Form of H_2_	H_2_‐loading capacity	Disease model	Administration route	References
Spontaneously responsive		PdH nanoparticle	H atoms	H_2_ gas	0.94 mmol g^−1^	Alzheimer's disease	Intracerebral injection	[89]
		TN‐PdH@Ms nanoparticle	H atoms	H_2_ gas	–	Atherosclerosis	Intravascular injection	[90]
		rGO@PdH nanoparticle	H atoms	H_2_ gas	–	Glioma U118 Cells	Cellular incubation	[91]
Internally responsive	Water responsive	Mg@p‐SiO_2_ MPs	H_2_O	H_2_ gas	39.5 mmol g^−1^	PC12 cells	Cellular incubation	[92]
		MgG rods	H_2_O	H_2_ gas	–	Tumor@@@(4T1, CT26, VX_2_)	Implantation	[93]
		CaH_2_ nanoparticle	H_2_O	H_2_ gas	–	Tumor@@@(4T1, CT26, VX_2_)	Intravenous injection	[94]
	Acid responsive	Fe@CMC nanoparticle	H^+^	H_2_ gas	9.5 mmol g^−1^	Tumor (4T1 cells)	Intravenous injection	[98]
		MBN@PVP	H^+^	H_2_ gas	62 mmol g^−1^	Gastric Cancer	Oral uptake	[99]
		mPDAB	H^+^	H_2_ gas	–	Tumor (4T1‐luc cells)	Intravenous injection	[100]
External responsive	Light responsive	AuNP@liposome nanoreactor	AA (H^+^)	H_2_ gas	–	LPS‐induced@@@inflammation	Local injection	[101]
		Pdots@liposome nanoreactor	AA (H^+^)	H_2_ gas	–	LPS‐induced@@@inflammation	Local injection	[102]
		[FeFe]TPP/GEM/FCS nanoparticle	H_2_O	H_2_ gas	13.6 µmol g^−1^ h^−1^	Bladder cancer	Intravesical instillation	[103]
	NIR responsive	PdH_0.2_ nanocrystal	H atoms	H_2_ gas	0.94 mmol g^−1^	Tumor@@@(4T1, B16‐F10, HeLa)	Intravenous injection	[107]
		PdH‐MOF nanoparticle	H atoms	H_2_ gas	4.7 mmol g^−1^	Tumor (4T1 cells)	Intravenous injection	[108]
		PdHs	H atoms	H_2_ gas	–	Tumor (4T1 cells)	Intratumor injections	[109]
		SnS_1.68_‐WO_2.41_ nanocatalyst	GSH (H^+^)	H_2_ gas	–	Tumor (4T1 cells)	Intravenous injection	[110]
		UCCZ nanoparticle	H_2_O	H_2_ gas	5.5 µM	Tumor (4T1 cells)	Intravenous injection	[111]
	Ultrasound responsive	H_2_‐MBs	H_2_	H_2_ gas	1.46 × 10^−3^ M	Myocardial ischemia‐reperfusion injury	Intravenous injection	[113]
		Pt‐Bi_2_S_3_	GSH (H^+^)	H_2_ gas	–	Tumor (4T1 cells)	Intravenous injection	[115]

### 3.1 Spontaneous Hydrogen Release

**Figure 4:**
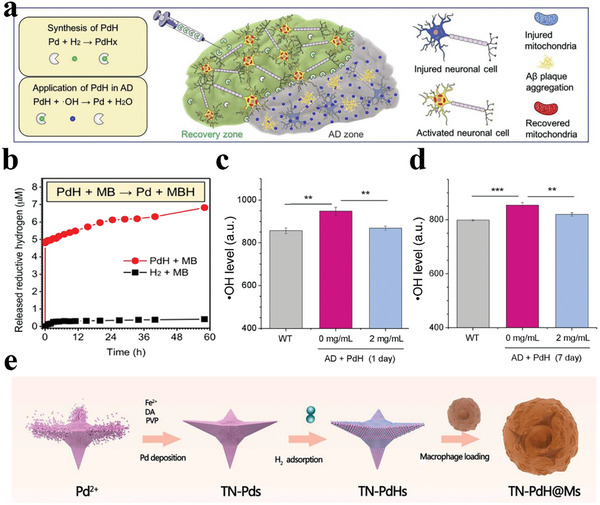
a) Schematic illustration of the strategies for the synthesis and anti‐AD application of PdH nanoparticles. b) H_2_ release profile of PdH nanoparticles. c,d) Effects of PdH nanoparticles (2 mg mL^−1^) on the level of ·OH in AD mice hippocampus for 1 day and 7 days. Reproduced with permission.^[^ ^89^ ^]^ Copyright 2019, Elsevier. e) Schematic illustration of synthetic procedure of TN‐PdH@Ms. Reproduced with permission.^[^ ^90^ ^]^ Copyright 2022, American Chemical Society.

In recent years, biomaterials with the ability of sustained H_2_ release have attracted much attention because of long‐term therapeutic H_2_ administration.^[^ ^85^, ^86^ ^]^ Palladium (Pd) usually serves as an excellent H_2_ storage material and an ideal hydrogenation catalyst in the field of energy, and its catalytic process is accompanied with stoichiometric generation of H_2_.^[^ ^87^, ^88^ ^]^ The chemical reaction of H_2_ generation during this process inspires biomedical researchers to employ Pd nanoparticles as H_2_ generator. Specifically, Pd nanocrystals can catalyze the active hydrogen atoms absorbed on their surface to form bio‐reductive H_2_ in a self‐catalysis way, thus realizing the spontaneous release of H_2_ gas. For example, Zhang et al. developed a small Pd hydride (PdH) nanoparticle with high H_2_ adsorption to treat AD (**Figure** **4**a).^[^ ^89^ ^]^ The synthesized PdH nanoparticles exhibited a cubic shape with a hydrodynamic diameter of ≈30 nm. They calculated that the H_2_ content in PdH had a H to Pd molar ratio of ≈0.2 (Figure 4b). After intracerebral injection, the PdH nanoparticles functioned as an *in‐situ* depot for the continuous production of bioactive H_2_, in which the process of H_2_ release was maintained by the catalytic hydrogenation effect of Pd. The released H_2_ possessed high bio‐reductivity, which could efficiently scavenge excessive ·OH in AD lesions, thereby blocking AD progression (Figure 4c,d).

A similar performance also occurs when the Pd nanoparticles change shape and size. Hu et al. used a distinct tetrapod needle‐like Pd nanoparticle as an H_2_ delivery platform to spontaneously release H_2_ gas for the efficient treatment of atherosclerosis (Figure 4e).^[^ ^90^ ^]^ The constructed tetrapod was ≈100 nm in size and had an edge length of nearly 60 nm. It was found that TN‐PdH could gradually release H_2_ gas due to its self‐catalysis effect. After being internalized inside macrophages, the H_2_ released from TN‐PdH exerted a strong inflammation‐inhibition effect to improve the environment within the artery walls, thereby alleviating atherosclerosis. In addition, Lan et al. synthesized reduced graphene oxide@Pd nanocomposites (rGO@PdH) to adsorb H_2_ for glioma treatment.^[^ ^91^ ^]^ After doping Pd nanospheres (4–20 nm) with porous graphene, the large specific surface area of graphene could evenly disperse the Pd and further improve the H_2_ storage capacity through a significant spillover effect. They used U118 glioma cells as a cell model to explore the effect of rGO@PdH on the redox level, cell proliferation, migration, and apoptosis. The results showed that the rGO@PdH composite nanomaterials could affect the redox level in glioma U118 cells, reducing their proliferation and migration ability.

Although nanoscale Pd materials have been verified to be an effective way to storage and release H_2_ for disease treatment, the effect of different sizes and shapes of Pd nanoparticles on their H_2_ adsorption capacity and their intrinsic interactions need to be further identified and investigated. Additionally, it is noticeable that the release of H_2_ molecules from Pd nanoparticles is mainly a spontaneous behavior, which lacks targeting and controllability, limiting the therapeutic effect of H_2_ to a certain extent. Thus, more strict strategies to control H_2_ generation are highly desired.

### 3.2 Internal Stimuli‐Responsive Hydrogen Release

Unlike biomaterials that release H_2_ spontaneously, internal stimuli‐triggered biomaterials utilize the difference in the chemical environment between disease tissues and normal sites to achieve the targeted H_2_ release and accumulation. With endogenous stimuli‐responsive strategies, the release rate and concentration of H_2_ can be effectively controlled to improve the therapeutic efficacy and reduce side effects. More importantly, this release mode is not limited by the penetration depth and is more favorable for tissue specific H_2_ therapy. Several typical examples are introduced as follows:

#### 3.2.1 Water Stimuli‐Response

**Figure 5:**
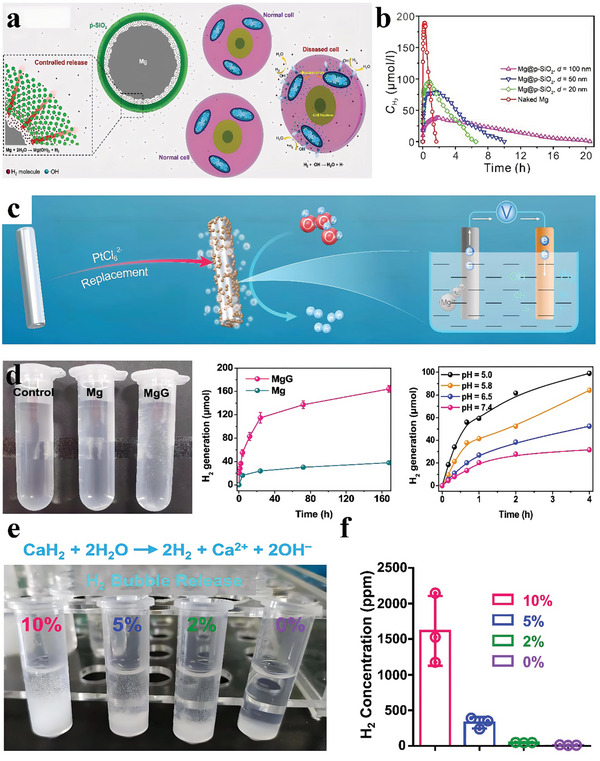
a) Schematic demonstration of the long‐term elimination of ·OH continuously released from diseased cells by Mg@p‐SiO_2_ MPs in body fluids. b) H_2_ release curves of naked Mg and Mg@p‐SiO_2_ MPs with different silica shell thicknesses. Reproduced with permission.^[^ ^92^ ^]^ Copyright 2018, Wiley‐VCH. c) Schematic illustration of the synthesis process of Mg galvanic cells and the H_2_ production mechanism. d) MgG rods for water‐triggered H_2_ release. Photograph showing H_2_ generation in PBS from Mg and MgG. Time‐dependent H_2_ generation from Mg or MgG rods in PBS. H_2_ generation profiles of MgG rods in PBS solutions with different pH values. Reproduced with permission.^[^ ^93^ ^]^ Copyright 2022, Nature Publishing Group. e) A photograph of H_2_ bubble generation in various solutions containing different ratios of H_2_O. f) The generated H_2_ concentration in various solutions as indicated via gas chromatography. Reproduced with permission.^[^ ^94^ ^]^ Copyright 2021, Elsevier.

Different from the above‐mentioned H_2_ storage materials, several active metals can react with water to produce stoichiometric H_2_. Among them, magnesium (Mg) usually serves as an H_2_ supplier for H_2_ generation in industries. In fact, too fast Mg degradation and the rapid generation of H_2_ limited its application for intracellular H_2_ therapy because H_2_ bubbles may cause mechanical damage to tissue repair and also bring a potential embolism risk. Therefore, slow and sustained Mg degradation and H_2_ release are highly desired. Recently, Kong et al. synthesized magnesium@mesoporous SiO_2_ core‐shell microparticles (Mg@p‐SiO_2_ MPs) by a modified Stöber method using acetone as the solvent (**Figure** **5**a).^[^ ^92^ ^]^ The Mg@p‐SiO_2_ MPs supplied H_2_ through the continuous Mg‐water reaction, in which the mesoporous SiO_2_ nanoshell thickness regulated H_2_ release. With the increase in SiO_2_ nanoshell thickness, the H_2_ release rate was decreased; however, the H_2_ release duration was significantly prolonged due to the barrier effect on the occurrence of Mg‐water reaction and H_2_ diffusion (Figure 5b). The sustained generation of H_2_ could eliminate ·OH in a biological medium over the long term to protect PC12 cells from oxidative damage. More importantly, most of the released H_2_ molecules could dissolve in the physiological medium without forming potentially harmful bubbles. To further increase the amount of H_2_ produced by the Mg‐water reaction, Yang et al. developed an Mg‐based galvanic cell (MgG), in which platinum nanoparticles decorated the surface of Mg rods (Figure 5c).^[^ ^93^ ^]^ In contrast to the electrolytic cells, the prepared MgG could be etched by water to spontaneously generate H_2_ gas without an external power supply. In detail, once placed in an aqueous solution, the Mg‐Pt galvanic cells underwent a spontaneous redox reaction. From the negative electrode (Mg electrode, Mg‐2e^−^ = Mg^2+^), electrons (e^−^) would flow into the positive electrode (Pt electrode, 2H_2_O+2e^−^ = 2OH^−^+H_2_), resulting in water etching of Mg and H_2_ generation (Figure 5d). The continuous generation of H_2_ could induce intracellular mitochondrial dysfunction and redox homeostasis destruction to achieve tumor treatment.

In addition to Mg‐based materials, calcium (Ca)‐based materials are also considered as portable H_2_ sources, which efficiently react with water to provide H_2_. For example, Gong et al. used the liquid‐phase exfoliation method to synthesize calcium hydride (CaH_2_) nanoparticles, followed by dispersal into low‐molecular‐weight polyethylene glycol (PEG) for synergistic H_2_‐immune cancer therapy.^[^ ^94^ ^]^ Abundant H_2_ gas and calcium ions (Ca^2+^) were generated when Nano‐CaH_2_ reacted with water, in a water content‐dependent manner (Figure 5e,f). The released H_2_ could trigger H_2_ therapy and promote anti‐tumor efficacy.

Compared with physically driven spontaneous H_2_ release, the generation amount and duration of H_2_ via chemical reactions with water are much larger. Moreover, the rate of H_2_ release can be controlled by the rational design of biomaterials, such as encapsulating them into specific materials. However, since chemical reaction usually occurs on the interface, the reaction starts once the biomaterials come into contact with water, which requires the encapsulated materials to possess good controllability and high stability. Therefore, other endogenous stimuli should be developed to trigger H_2_ release from biomaterials.

#### 3.2.2 Acid Stimuli‐Response

The uncontrolled growth of tumor cells and abnormal gene expression result in tumor tissues exhibiting significantly different physiological characteristics from normal tissues. The weak acidity of the tumor microenvironment (TME) caused by a large amount of anaerobic glycolysis is a crucial characteristic of malignant tumors.^[^ ^95^, ^96^ ^]^ Therefore, the pH difference can be used as a stimulus for designing responsive biomaterials. Based on this, researchers have constructed a series of acid stimuli‐responsive biomaterials to release H_2_. Noticeably, H_2_ suppliers must remain stable under physiological conditions and only produce H_2_ under acidic conditions.

**Figure 6:**
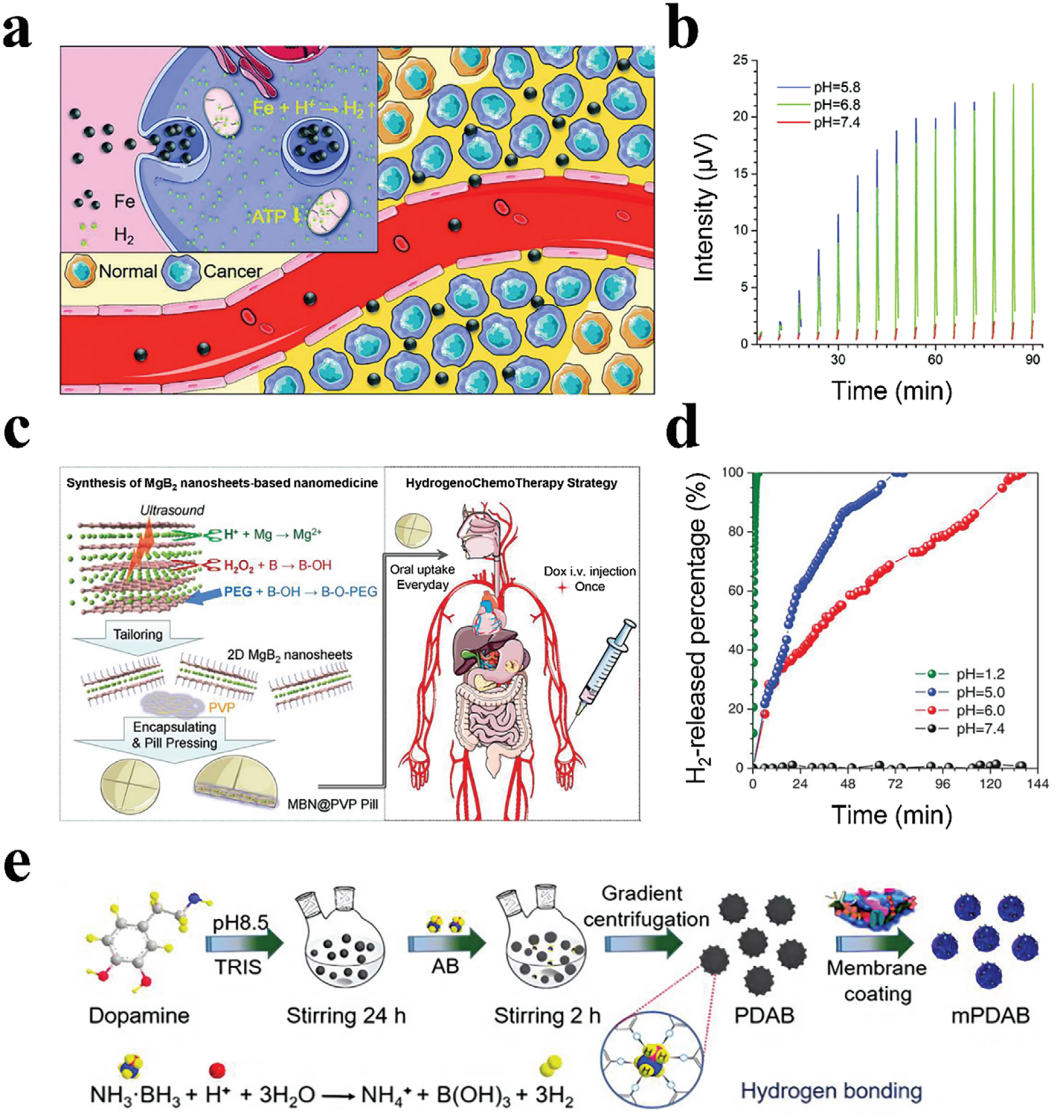
a) Schematic illustration of the mechanisms for the tumor‐targeted acid‐responsive release of H_2_. b) H_2_ release profiles of Fe@CMC nanoparticles at different pH values determined by gas chromatography. Reproduced with permission.^[^ ^98^ ^]^ Copyright 2019, Royal Society of Chemistry. c) Schematic illustration of the synthesis route and therapeutical strategy of MBN@PVP pills. d) H_2_ release profiles of MBNs in PBS solutions with different pH values. Reproduced with permission.^[^ ^99^ ^]^ Copyright 2019, Wiley‐VCH. e) Schematic illustration of mPDAB nanoparticles for tumor therapy. Reproduced with permission.^[^ ^100^ ^]^ Copyright 2019, Elsevier.

Iron is a biodegradable metal that is used widely in various medical devices and could potentially generate H_2_.^[^ ^97^ ^]^ Kou et al. successfully developed core‐shell nanoparticles with Fe as the core and sodium carboxymethyl cellulose (CMC) as the shell for tumor‐targeted acid‐responsive H_2_ release (**Figure** **6**a).^[^ ^98^ ^]^ The CMC coated on the Fe nanoparticle surface effectively protected Fe from oxidation in blood circulation. Their small size endowed Fe@CMC nanoparticles with a good passive targeting ability and high responsiveness to H_2_ production in a weak acid environment, which was conducive to intra‐tumoral H_2_ accumulation and effective H_2_ therapy. Experiments demonstrated that the Fe@CMC nanoparticles were relatively stable in phosphate‐buffered saline (PBS) at pH 7.4, with no H_2_ release; however, in PBS at pH 6.8, they showed sustained decomposition into H_2_ gas, demonstrating high sensitivity to acid resulting from the nano‐formula's high reactivity (Figure 6b). This acid‐triggered H_2_‐releasing strategy displayed high biosafety and good anticancer efficacy.

Besides iron materials, magnesium boride (MgB_2_) nanosheet is also considered as a kind of H_2_‐generating material that is chemically active enough to hydrolyze into H_2_ under acidic conditions (producing 3 mol H_2_ per mol MgB_2_). Fan et al. prepared polyethylene pyrrolidone (PVP)‐encapsulating 2D magnesium boride nanosheet (MBN) as an acid‐sensitive H_2_‐releasing nanoparticle using an ultrasound‐assisted chemical etching method (Figure 6c).^[^ ^99^ ^]^ The synthesized MBN@PVP was highly stable in normal tissues/blood circulation. Meanwhile, it showed high gastric acid sensitivity, enabling an acid‐responsive release of H_2_ gas in the stomach for up to 12 days to significantly enhance the therapeutic effect (Figure 6d). The developed H_2_‐releasing 2D nanomedicine provides a new pathway for H_2_ therapy.

In addition to preparing H_2_‐producing nanomaterials, another strategy is the construction of nanomedicine with an H_2_ prodrug and a nanocarrier. Ammonia borane (AB), an acid‐sensitive pro‐drug that can generate H_2_ or owns a high H_2_‐loading capacity, is rapidly hydrolyzed. Zhang et al. anchored AB onto polydopamine (PDA) nanosphere surfaces, which stabilized AB via H_2_ bonding (Figure 6e).^[^ ^100^ ^]^ The as‐prepared nanoparticles exhibited a highly acid‐responsive H_2_ release behavior and could react with H_2_ ions in an acid solution (pH 6.8), forming boron hydroxide and continuously releasing H_2_ gas. Notably, the AB‐mediated release of H_2_ was pH‐dependent.

In conclusion, these acid‐responsive H_2_‐releasing biomaterials notably increase the bioavailability of H_2_ in tumor cells and improve therapeutic efficacy. However, the current reactions mainly rely on the acidity of the cancer cell microenvironment, which limits the application of H_2_ therapy in other diseases. In addition, the accumulation of reaction products, such as OH^−^ may disrupt the electrolyte balance of tumor cells and affect the H_2_ therapeutic efficacy. These factors need to be considered in the future application of H_2_ therapy.

### 3.3 External Stimuli‐Responsive Hydrogen Release

Recently, external stimuli‐responsive strategies have received enormous interest due to their non‐invasiveness and practicability. The exogenous stimuli such as light and ultrasound have shown advantages of spatiotemporal controllability in drug response release, which may achieve on‐demand H_2_ release predictably. It is also easy to control the rate and amount of H_2_ release by adjusting the switching, energy, and irradiation time of the stimulus source. This section will detail biomaterials that are used for H_2_ therapy and are triggered by external stimuli.

#### 3.3.1 Visible Light Stimuli‐Response

**Figure 7:**
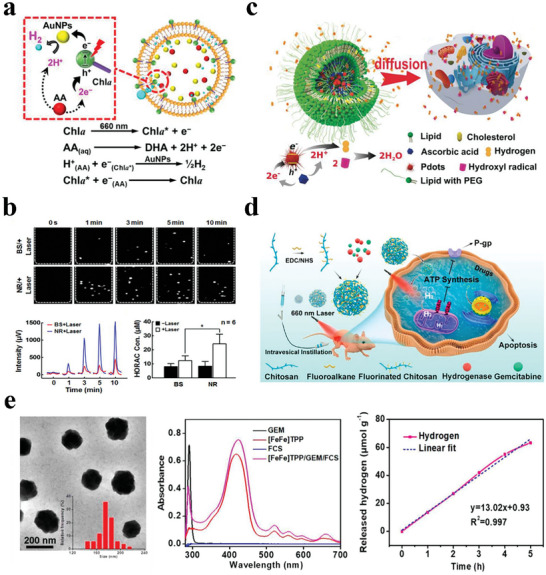
a) Structure and mechanisms of H_2_ generation by photosynthesis. b) H_2_ release profile and ·OH scavenging activities of AuNP@liposome nanoreactor without/with laser irradiation. Ultrasound images and concentrations of generated H_2_ in bulk solution and nanoreactor after laser irradiation. ·OH scavenging activities of bulk solution and nanoreactor without/with laser irradiation. Reproduced with permission.^[^ ^101^ ^]^ Copyright 2017, American Chemical Society. c) Schematic illustration of liposomal compartment as nanofactory in intercellular space for in situ photocatalytic H_2_ generation to scavenge ·OH. Reproduced with permission.^[^ ^102^ ^]^ Copyright 2019, Wiley‐VCH. d) Schematic of the in situ photoactivated H_2_ nanogenerator for enhanced chemotherapy of bladder cancer. e) [FeFe]TPP/GEM/FCS NPs for light‐triggered H_2_ release. TEM image and hydrodynamic diameters of [FeFe]TPP/GEM/FCS NPs. UV–vis absorbance spectra of different formulations. Linear fit of the H_2_ evolution experimental results. Reproduced with permission.^[^ ^103^ ^]^ Copyright 2020, American Chemical Society.

For on‐demand drug administration, light‐induced drug delivery represents a facile method. Photocatalytic water splitting to control H_2_ release provides a new method for H_2_ therapy. Wan et al. designed a multicomponent nanoreactor to generate H_2_ locally (**Figure** **7**a).^[^ ^101^ ^]^ The nanoreactor utilized a liposome‐encapsulated photosensitizer (chlorophyll a; Chla), a proton donor (AA), and a catalyst for H_2_ production (gold nanoparticles; AuNPs). Under the 660 nm laser irradiation, Chla produced electrons and oxidation of AA produced protons, which were collected by the AuNPs, thus promoting their conversion to H_2_ gas. Experimental results showed that the concentration of released H_2_ using this artificial photosynthesis was proportional to the duration of light exposure (Figure 7b). It is expected that sustained H_2_ release could be regulated by adjusting the light time and controlling the light switch, thus achieving long‐term H_2_ therapy.

In addition to using metal catalysts to realize light triggered H_2_ release, non‐metal photocatalysts can also be used. Zhang et al. selected semiconducting polymer dots (Pdots) with photocatalytic activity and biocompatibility for H_2_ generation (Figure 7c).^[^ ^102^ ^]^ The nanoreactor comprised AA and the Pdot photocatalyst encapsulated in a liposome. When the Pdots absorbed photons, the catalytic cycle started to produce H_2_ within the aqueous lumen. The released H_2_ could diffuse into the lesion and effectively reduce the inflammatory responses induced by lipopolysaccharide (LPS).

Inspired by metalloenzymes, Sun et al. developed a photoactivated H_2_ nanogenerator comprising a catalyst for H_2_ production ([FeFe]TPP), a chemotherapeutic agent (gemcitabine, GEM), and fluorinated chitosan (FCS), which could self‐assemble into nanoparticles ([FeFe]TPP/GEM/FCS NPs) (Figure 7d).^[^ ^103^ ^]^ When irradiated using a 660 nm laser, the as‐prepared nanoparticles could efficiently decompose water into H_2_ gas in situ in an irradiation time‐dependent manner (Figure 7e). After intravesical instillation into the bladder, the produced H_2_ from [FeFe]TPP/GEM/FCS NPs exhibited excellent therapeutic efficacy and safety to treat bladder cancer.

Although visible light induced active H_2_ release from biomaterials has shown great potential for disease treatment, the production amount of H_2_ from these systems is limited by their photocatalyst loading capacity. Furthermore, the low tissue penetration depth of visible light also is an obstacle to the applications of H_2_ therapy. Therefore, it is necessary to develop other stimuli‐responsive biomaterials for H_2_ release in inner tissues.

#### 3.3.2 Near‐Infrared (NIR) Stimuli‐Response

**Figure 8:**
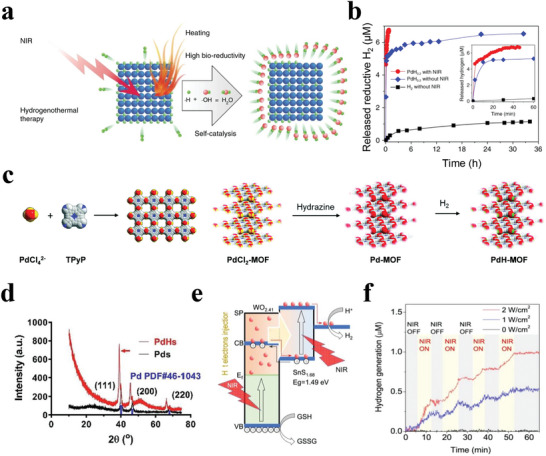
a) Schematic illustration of NIR‐responsive H_2_ release mechanism of PdH_0.2_ nanocrystals. b) H_2_ release from PdH_0.2_ nanocrystals in stimulated body fluid. Reproduced with permission.^[^ ^107^ ^]^ Copyright 2018, Nature Publishing Group. c) Schematic of the synthesis process of PdCl_2_‐MOF. Reproduced with permission.^[^ ^108^ ^]^ Copyright 2019, Royal Society of Chemistry. d) PdHs for NIR‐triggered H_2_ release. Reproduced with permission.^[^ ^109^ ^]^ Copyright 2022, Elsevier. e) Schematic illustration of the mechanism for NIR‐photocatalytic H_2_ generation of SnS_1.68_‐WO_2.41_ nanocatalyst. f) NIR‐photocatalytic H_2_ generation behavior of the SnS_1.68_‐WO_2.41_ nanocatalyst under irradiation of 808 nm laser at various power densities. Reproduced with permission.^[^ ^110^ ^]^ Copyright 2021, Nature Publishing Group.

Light in the NIR region (700–1700 nm) can penetrate deeper into the tissue, scatter less, and cause minimal tissue damage compared with visible light (400–700 nm).^[^ ^104^ ^]^ Importantly, NIR light is converted to heat energy by NIR photothermal agents to allow temperature‐responsive drug release. Therefore, light‐dependent drug delivery preferentially uses an NIR laser.^[^ ^105^, ^106^ ^]^ In this section, we summarized two strategies for the design and construction of NIR‐responsive H_2_‐releasing biomaterials; one is the direct synthesis of NIR‐responsive nanoparticles by using the Z‐scheme structure of nanoparticles to convert the photoelectronic energy into photochemical energy for NIR‐responsive H_2_ release. Zhao et al. used Pd nanoparticles as a carrier to synthesize Pd hydride (PdH_0.2_) nanocrystals using the H_2_ bubbling method to enhance photothermal therapy (PTT) (**Figure** **8**a).^[^ ^107^ ^]^ NIR laser irradiation destroyed the Pd‐H binding force to release active H_2_, the rate of which depended on the conversion properties of the Pd nanoparticles. Pt‐microelectrode methods, an MB probe, and UV absorption spectroscopy were used to investigate the mechanism by which H_2_ has released responsive NIR. The results revealed that the NIR irradiation could excite PdH_0.2_ to release H_2_ gas. The H_2_‐incorporation capacity of the Pd nanocrystals was calculated to be ≈0.2 mol mol^−1^ (Figure 8b). Similarly, PdH_0.2_ was responsive to NIR light in a power density‐dependent manner. To further increase the H_2_ loading, Zhou et al. constructed a nanoscale porphyrin‐palladium metal–organic framework (Pd‐MOF) containing highly dispersed Pd atoms, which acted as H_2_ carriers for hydrogenothermal cancer therapy (Figure 8c).^[^ ^108^ ^]^ The uniform dispersion of single‐atom Pd in the MOF meant that it adsorbed H_2_ gas more easily. Similarly, the local temperature increase induced by NIR irradiation disrupted the Pd‐H binding force, thereby triggering H_2_ release. According to the experimental results, in Pd‐MOF, the ratio of H to Pd was ≈1, which was much higher than that of previously reported PdH_0.2_ nanocrystals. The Pd‐MOFs had a high capacity for H_2_ loading and displayed sustained H_2_ release, making a strong contribution to H_2_ therapy.

A better therapeutic effect can be realized through structural and functional design. Lin et al. employed a two‐step hydrothermal method to construct Pd hydride nanourchins (PdHs) for usage in photothermal immunotherapy (Figure 8d).^[^ ^109^ ^]^ The unique dendritic urchin structures of PdHs presented significantly enhanced H_2_ release when irradiated using an 808 nm laser. With the increased NIR irradiation time, the absorbance of PdHs shifted gradually toward Pd, suggesting the escape of the H atoms as H_2_ gas from the PdH lattice. The controlled H_2_ release further caused increased ROS and oxidative stress to realize enhanced PTT.

NIR‐driven H_2_ evolution photocatalysts represent another strategy for H_2_ therapy. Jin et al. designed a Z‐scheme structure by in situ conjugation of WO_3‐x_ nanodots (highly oxidative and plasmatic) onto SnS_2‐y_ nanoplates (NIR‐activable semiconductors) to realize NIR‐photocatalytic H_2_ generation for cancer treatment (Figure 8e).^[^ ^110^ ^]^ This heterojunction could ensure that NIR‐driven hot electrons from WO_2.41_ nanodots were injected into the conjugated SnS_1.68_ nanoplates, thus generating H_2_ via enhanced NIR‐photocatalytic reduction. Under NIR irradiation, electrons (e^−^) and holes (h^+^) were produced and separated on the surface of the SnS_1.68_ nanoplates and WO_2.41_ nanodots, respectively. More specifically, on the WO_2.41_ nanodot surface, h^+^ oxidized GSH simultaneously generated H^+^. The H^+^ ions were transferred to the SnS_1.68_ nanoplates and reduced to H_2_ by e^−^. The results showed that the nanocatalyst could generate H_2_ gas under NIR excitation, the rate of which depended markedly on the power density of the NIR laser. More importantly, the NIR laser could be switched on or off to control and repeat the process, thereby achieving on‐demand H_2_ therapy (Figure 8f).

In addition to photocatalysts, photo‐converting materials, particularly upconversion nanoparticles (UCNPs) can also be applied for NIR controlled H_2_ release by converting NIR light (*λ* = 980 nm) into UV/Vis light. Wang et al. prepared a nanocomposite (NaGdF4: Yb, Tm/g‐C_3_N_4_/Cu_3_P) to generate H_2_ by splitting water through 980 nm laser irradiation for cancer therapy.^[^ ^111^ ^]^ First, this nanostructure could convert NIR light into visible light, which excited g‐C_3_N_4_/Cu_3_P to form electrons and holes. Then, the photogenerated electrons with higher reducibility in the conduction band (CB) of Cu_3_P reduced water to H_2_. The results showed that this heterojunction possessed an enhanced photocatalytic H_2_ production ability, which released H_2_ continuously up to ≈5.5 µM.

The above studies confirmed that NIR‐responsive H_2_ release based on biomaterials is an effective way for local therapy. However, NIR‐responsive materials usually possess photothermal effects, and the elevated temperature may cause damage to normal tissues, thus affecting the efficacy of H_2_ therapy. In addition, synergistic treatment with photothermal and hydrogen therapies may induce complex mechanisms and unclear molecular functions. These intrinsic factors should be further considered in NIR‐triggered H_2_ therapy.

#### 3.3.3 Ultrasound (US) Stimuli‐Response

**Figure 9:**
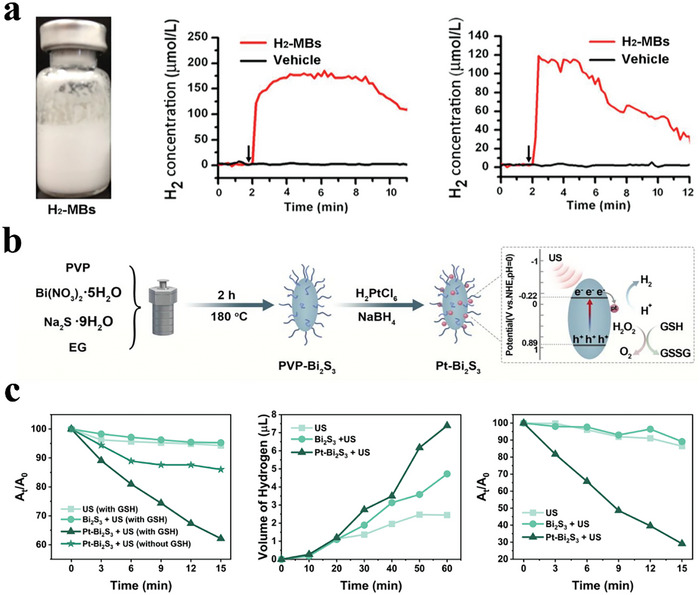
a) Representative image of the H_2_‐MBs. Representative time courses of the H_2_ release in vitro and in vivo. Reproduced with permission.^[^ ^113^ ^]^ Copyright 2017, American Chemical Society. b) Diagram showing the synthetic process and mechanism of H_2_ generation from Pt‐Bi_2_S_3_. c) US‐triggered H_2_ release profile of Pt‐Bi_2_S_3_. Relative absorption value at 665 nm of the different formulations. The produced H_2_ volume of different groups. GSH consumption rate of different groups. Reproduced with permission.^[^ ^115^ ^]^ Copyright 2022, Wiley‐VCH.

As another non‐invasive stimulus source, the US has gained increasing research attention due to its safety (no radiation), high tissue contrast, real‐time results, non‐invasive nature, and low‐cost.^[^ ^112^ ^]^ Since the FDA approved the first generation of microbubbles, AlbunexR, the biocompatibility and stability of US‐based contrast agents have evolved rapidly. Under the influence of the US, drugs can be released from US‐responsive systems. For example, He et al. constructed an ultrasound‐aided system to deliver H_2_ by loading reduced H_2_ gas into microbubbles (H_2_‐MBs) for the treatment of myocardial ischemia‐reperfusion injury (**Figure** **9**a).^[^ ^113^ ^]^ The H_2_‐MBs accumulated at the lesion site according to their enhanced permeability and retention (EPR) effect and could be visually tracked using ultrasound imaging systems.^[^ ^114^ ^]^ The authors showed that the US signal intensity was directly proportional to the H_2_‐MBs concentration. Thus, the therapeutic H_2_ concentration could be regulated by adjusting the US intensity. Furthermore, the ultrasound imaging capability of this system provided an opportunity to monitor H_2_ gas in real‐time. This strategy showed promise as a visual release system for therapeutic applications based on H_2_.

In addition to constructing microbubbles as H_2_ carriers, another strategy is to directly synthesize nanosized H_2_‐generating materials. For instance, Yuan et al. designed platinum‐bismuth sulfide heterostructure nanocomposites (Pt‐Bi_2_S_3_) as sonocatalysts for H_2_ therapy (Figure 9b).^[^ ^115^ ^]^ Under US excitation, the produced electrons transferred from the surface of Bi_2_S_3_ nanorods to the Fermi energy level of Pt nanoparticles through Schottky junctions. Meanwhile, sono‐excited holes in the valence band of Bi_2_S_3_ nanorods were consumed by the natural hole sacrificial agent GSH. The combined effect improved the charge separation efficiency and promoted long‐term H_2_ generation (Figure 9c). This nanocatalytic system showed good H_2_ production performance and induced apoptosis of tumor cells by inhibiting intracellular energy metabolism and destroying the tumor antioxidant defense system. Therefore, the US may serve as a promising trigger to control H_2_ delivery and release for specific disease treatment.

## 4 Biomaterial‐Based Hydrogen Therapy Strategies and Applications

**Figure 10:**
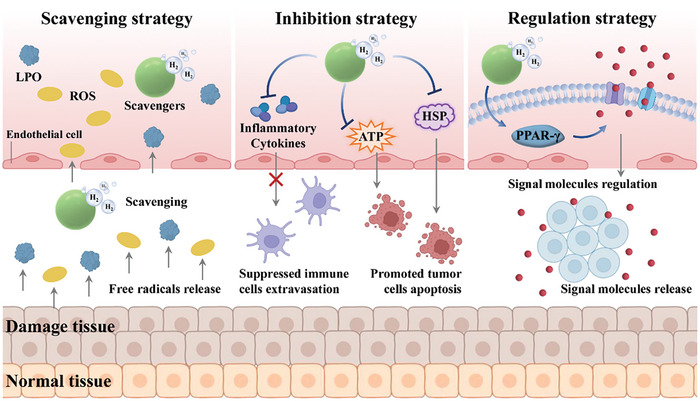
Overview of predominantly regulated mechanisms of biomaterial‐based H_2_ therapy. Biomaterial‐based H_2_ therapy can be applied to treat diseases by free radicals scavenging, danger factors inhibition, and signal molecule regulation. Figure Created through BioRender.com.

Recently, biomaterial‐based stimuli‐responsive H_2_ release strategies based on their precise, controllable, and targeted H_2_ release, have been developed to treat various diseases. According to the pathogenesis and characteristics of diseases, strategies for H_2_ therapy based on biomaterials can be divided into the following three categories: free radicals scavenging strategy, danger factors inhibition strategy, and signal molecules regulation strategy (**Figure** **10**).

### 4.1 Scavenging Strategies

#### 4.1.1 ROS Scavenger

ROS are signal molecules or metabolites generated by organisms in the process of cellular metabolism or under environmental stimulation. Low levels of ROS can act as cellular signal messengers to modify protein structure and function by reversibly oxidizing thiol groups in them. However, high concentrations of ROS can disrupt cellular processes by non‐specifically attacking DNA, lipids, and proteins.^[^ ^116^ ^]^ Finally, the excessive ROS produced by cells can cause oxidative stress, thus leading to the occurrence of many diseases. For instance, ROS can regulate the expression of specific proteins involved in cell proliferation and metastasis by activating pro‐oncogenic signaling pathways, thereby accelerating tumor progression.^[^ ^117^, ^118^ ^]^ ROS can also induce endoplasmic reticulum (ER) stress and disrupt ER function, leading to sustained Ca^2+^ release, which consequently causes various inflammation‐related pathological abnormalities and even neurodegeneration.^[^ ^119^, ^120^, ^121^ ^]^ In this case, modulation of ROS concentrations by antioxidant therapy can maintain intracellular redox homeostasis and prevent oxidative stress‐related diseases.^[^ ^122^, ^123^ ^]^

ROS can be maintained within a reasonable range by certain antioxidant enzymes, such as SOD, CAT, and glutathione peroxidase (GPx).^[^ ^124^, ^125^ ^]^ At the lesion sites, the levels of these antioxidant enzymes are relatively low due to the unbalanced redox status, thus exhibiting weak ROS‐eliminating abilities. However, direct supplementation with natural antioxidant enzymes usually has limited therapeutic effects on the disease because natural enzymes tend to have specific antioxidant capacities and hardly scavenge multiple ROS simultaneously. In addition, natural antioxidant enzymes are easily degraded in vivo and have poor stability. As previously mentioned, H_2_ can selectively scavenge various highly toxic ROS without affecting the normal physiological activity of free radicals. Moreover, the antioxidant ability of H_2_ is stable. Owing to these properties, H_2_ therapy can effectively inhibit oxidative stress for disease treatment. However, due to the rapid diffusion of H_2_ gas, the H_2_ content in the lesions will decrease quickly, failing to maintain the effective concentration for a long time, which severely restricts the application of H_2_ in oxidative damage‐related diseases. With the remarkable development of responsive biomaterials, H_2_‐releasing biomaterials can achieve efficient and stimulus‐controlled long‐term delivery of H_2_ in diseased tissues. Recently, these systems have been widely studied for ROS‐associated disease treatment with satisfactory therapeutic effects.

**Figure 11:**
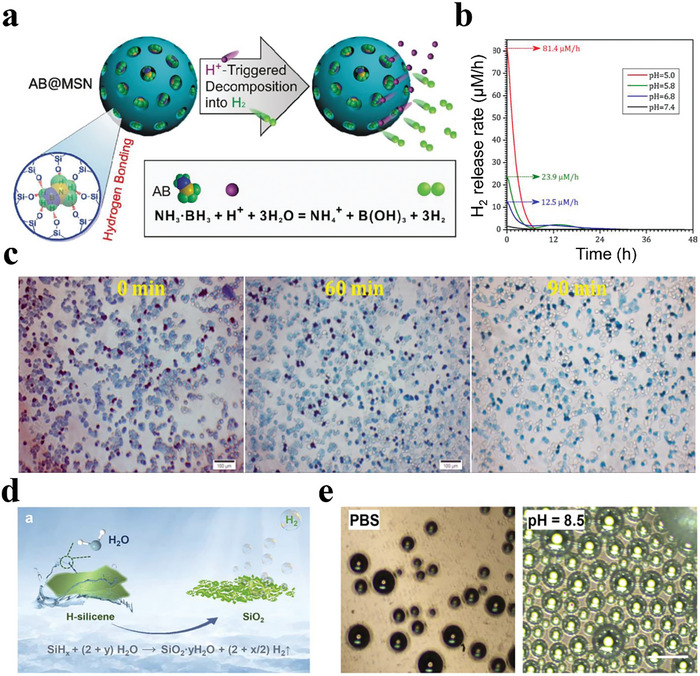
a) Schematic illustration of acid‐triggered hydrogen release from AB@MSN. b) The H_2_ release behaviors of AB@MSN nanomedicine. c) Qualitative observation of blue reduction with the intracellular hydrogen release of AB@MSN nanomedicine. Reproduced with permission.^[^ ^126^ ^]^ Copyright 2018, Elsevier. d) Schematic illustration of H_2_ generation from H‐silicene. e) Optical microscopic images of H_2_ bubble generation from H‐silicene during reaction with PBS and NaOH solution (pH 8.5). Reproduced with permission.^[^ ^127^ ^]^ Copyright 2022, American Chemical Society.

For tumor treatment, He et al. used mesoporous silica (MSN) loaded with AB to synthesize a nanomedicine (AB@MSN) that could release H_2_ gas in situ under the control of acid (**Figure** **11**a).^[^ ^126^ ^]^ MSN has a high AB‐loading capacity (653 mg g^−1^) because of its large surface area and has good biocompatibility, resulting in very high H_2_ loading (130.6 mg g^−1^) (Figure 11b). The sustained release of H_2_ from AB@MSN exerted selective antioxidant effects that led to tumor cell injury and apoptosis by disrupting redox homeostasis within the tumor, including reducing highly expressed ROS in tumor cells (Figure 11c). Meanwhile, the exogenous H_2_ could eliminate the negative effect of over‐quick release from normal cells. The results showed that this nanomedicine‐based H_2_ therapy strategy had good anticancer effects. However, the accumulation of OH^−^ ions or other productions during this chemical reaction might break the electrolyte balance of cells, undergoing apoptosis.

For oxidative stress‐induced inflammation, Shi et al. discovered that an H_2_‐terminated silicon nanosheet (H‐silicene) could react with water, rapidly and continuously generating H_2_ in the absence of external energy input to treat acute inflammation (Figure 11d).^[^ ^127^ ^]^ Inspired by the unique physical and chemical properties of emerging 2D nanomaterials, they improved silicon materials in terms of scale, structure, and covalent modification to circumvent the kinetic barrier associated with the traditional silicon‐water reaction. The prepared ultrathin 2D H‐silicene nanosheets had a large specific surface area and many active surface Si─H bonds, which could release H_2_ more efficiently in the physiological environment (Figure 11e). The generated gaseous H_2_ could scavenge intracellular ROS in a dose‐dependent pattern, thus protecting different cells from oxidative stress‐induced damage. Inspired by the antioxidant property of H‐silicene, two types of mice models of acute inflammation (ear swelling and paw inflammation) were constructed to explore their therapeutic effect. The in vivo experiments demonstrated that H‐silicene could act as an H_2_ supplier to rapidly relieve ROS‐induced inflammation, which provided an effective strategy for H_2_‐mediated disease treatment.

**Figure 12:**
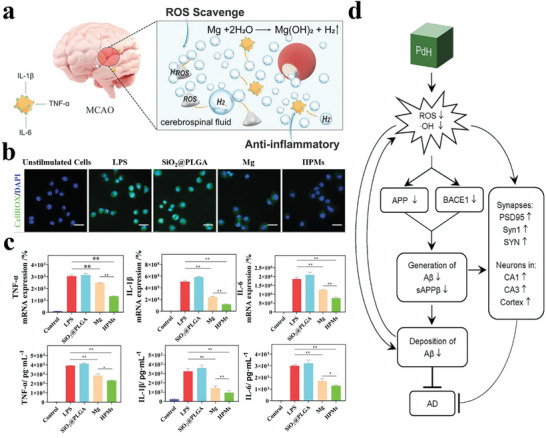
a) Schematic illustration of HPMs as in situ H_2_ generators for ischemic stroke remedy by active H_2_ delivery to scavenge ROS and alleviate oxidative stress. b) CLSM images of intracellular ROS after different treatments. c) mRNA expression and levels of TNF‐α, IL‐1β, and IL‐6 in LPS‐induced RAW264.7 cells after different treatments. Reproduced with permission.^[^ ^128^ ^]^ Copyright 2021, Wiley‐VCH. d) Schematic illustration of the anti‐AD mechanism for PdH reducing Aβ generation and deposition and ameliorating synaptic deficit and neuronal death by scavenging ·OH. Reproduced with permission.^[^ ^89^ ^]^ Copyright 2019, Elsevier.

According to previous reports, the pathogenesis of CNS diseases is mainly due to the excessive production of ROS caused by cerebral ischemia. Thus, scavenging ROS has been an effective method for CNS disease treatment. For example, Tu et al. fabricated H_2_‐powered microswimmers (HPMs) by coating a biodegradable polylactic acid‐glycolic acid copolymer (PLGA) layer onto Mg microparticles to treat acute ischemic stroke (**Figure** **12**a).^[^ ^128^ ^]^ The H_2_ generated locally from the reaction between HPMs and water not only acted as a motion propellant but also could effectively eliminate ROS (Figure 12b). Meanwhile, the autonomous movement of HPMs could further control H_2_ release and enhance diffusion, realizing active H_2_ delivery and improving intracellular reduction. A stereotaxic apparatus device was used to inject the HPMs precisely into the lateral ventricle of rats with middle cerebral artery occlusion (MCAO). After treatment, the HPMs could reduce the infarct volume and improve spatial learning and memory ability by scavenging ROS and inflammation (Figure 12c). This study is the first report that micromotors are used for actively scavenging ROS, which provides a new direction for acute ischemic stroke treatment. However, the H_2_ delivery of HPMs to lesion tissues was based on the specific device, which also restricted their efficacy and application in other diseases.

In addition, there have been many studies on the treatment of AD using H_2_.^[^ ^129^, ^130^, ^131^, ^132^ ^]^ To improve the therapeutic effect of H_2_ on AD, Zhang et al. designed small‐sized PdH nanoparticles with a high H_2_ payload that produced sustained in situ release of H_2_ at AD lesions.^[^ ^89^ ^]^ The catalytic hydrogenation profile of Pd led to the PdH nanoparticles releasing bio‐reductive H_2_ self‐catalytically, which selectively removed highly cytotoxic ·OH. Furthermore, the reduced ROS level inhibited the expression of amyloid precursor protein (APP) and β‐secretase (BACE1) to decrease the overproduction of Aβ in mice with AD, thus limiting AD progression (Figure 12d). Moreover, the released H_2_ could ameliorate mitochondrial dysfunction, promote energy metabolism in neurons, and block apoptosis by abrogating oxidative stress and activating anti‐oxidative pathways. After treatment with PdH nanoparticles, the mice with AD showed a remarkable improvement in cognition. Nevertheless, the high density of Pd led to its low unit mass storage capacity of H_2_. The biological toxicity of Pd is another issue to be considered.

#### 4.1.2 LPO Scavenger

LPO, a kind of free radical, is highly associated with oxidative damage, where overproduced ROS can destroy cells by inducing LPO.^[^ ^133^ ^]^ In this case, scavenging LPO with H_2_ is a promising method for disease treatment. As previously mentioned, Hu et al. developed tetrapod needle‐like PdH nanozymes (TN‐PdHs) to attenuate atherosclerosis.^[^ ^90^ ^]^ The TN‐PdHs were loaded into macrophages, which affected the targeted delivery of the TN‐PdHs to arterial plaques, where the spontaneous release of H_2_ from TN‐PdHs exhibited antioxidant and anti‐inflammatory effects to eliminate ROS and inhibit the expression of inflammatory cytokines, thus inhibiting atherosclerosis development. Notably, TN‐PdHs also could decrease the LPO level in a dose‐dependent manner, and ≈60% of the LPO was scavenged by 125 µg mL^−1^ TN‐PdHs. The in vitro and in vivo experiments demonstrated that the TN‐PdHs efficiently reduced the accumulation of intracellular lipids and prevented the formation of foam cells to treat atherosclerosis.

There is no doubt that H_2_ is effective because of its selective free radical scavenging properties, which have been confirmed in the treatment of many diseases. However, there has been limited information on the processes and specific molecular mechanisms of H_2_ therapy in regulating physiological/pathological pathways in vivo, and further research and elucidation are needed.

### 4.2 Inhibition Strategies

#### 4.2.1 Inhibition of Pro‐Inflammatory Cytokines

Inflammation is associated with a variety of acute and chronic diseases such as osteoarthritis, sepsis, asthma, etc. All these diseases involve the overproduction of pro‐inflammatory cytokines, which can cause persistent immune responses and eventually lead to degeneration at inflammatory lesions. Specifically, pro‐inflammatory cytokines can induce differentiation and proliferation of massive immune cells and disturb normal cell functions, thus promoting inflammation progression.^[^ ^134^, ^135^ ^]^ Therefore, inhibiting the production of pro‐inflammatory cytokines at the inflammatory sites can attenuate inflammation. Encouraged by the previously mentioned anti‐inflammatory effect of H_2_ molecules, biomaterial‐based H_2_ therapy is designed to regulate inflammation selectively. The released H_2_ from biomaterials can produce a marked effect by inhibiting or blocking the excessive pro‐inflammatory cytokines in inflammatory lesions.

**Figure 13:**
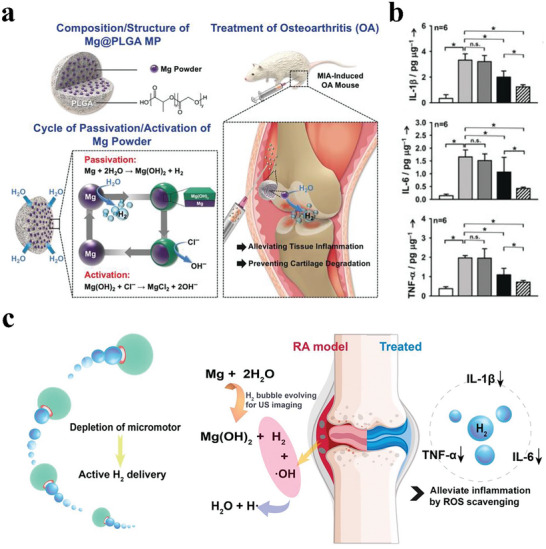
a) Schematic illustration of local injection of Mg@PLGA microparticles for the treatment of osteoarthritis. b) Levels of pro‐inflammatory cytokines IL‐1β, IL‐6, and TNF‐α in knee joints following various treatments. Reproduced with permission.^[^ ^136^ ^]^ Copyright 2018, Wiley‐VCH. c) Mechanism of the H_2_‐mediated RA curative effect by Mg‐HA motors. Reproduced with permission.^[^ ^137^ ^]^ Copyright 2021, American Chemical Society.

Inspired by this, Wan et al. prepared poly (lactic‐co‐glycolic acid) microparticles containing magnesium powder (Mg@PLGA MPs) to produce and sustainably release H_2_ gas for inflammation treatment (**Figure** **13**a).^[^ ^136^ ^]^ The Mg@PLGA MPs continuously produced H_2_ via a cycle of passivation/activation of Mg in body fluids. The rate of H_2_ release was controlled by the hydrophobic PLGA, which probably impeded the infiltration of water into the Mg@PLGA MPs. Cell experiments showed that Mg@PLGA MPs could significantly suppress the inflammatory responses of LPS‐stimulated macrophages. After intramuscular injection of Mg@PLGA MPs into a knee with osteoarthritis (OA), the generated H_2_ could reduce tissue inflammation and prevent the destruction of cartilage by effectively decreasing pro‐inflammatory cytokine levels (IL‐1β, IL‐6, and TNF‐α) and inhibiting infiltration of inflammatory cells, thereby arresting the development of OA (Figure 13b). However, the therapy efficacy of local H_2_ would be influenced by many factors, including the design of microfactories, the microenvironment of cells, and the properties of reaction products.

To expand the application of H_2_ inflammation therapy, Xu et al. fabricated a self‐propelled magnesium micromotor coated with hyaluronic acid (Mg‐HA motor) to precisely treat rheumatoid arthritis (RA) (Figure 13c).^[^ ^137^ ^]^ The Mg‐water reaction by the Mg‐HA motors drove the continuous generation of H_2_. The released active H_2_ exerted an outstanding capability to mitigate the inflammation responses by simultaneously down‐regulating the expression levels and eliminating the concentrations of pro‐inflammatory cytokine (IL‐1β, IL‐6, and TNF‐α). In a rat model of collagen‐induced arthritis, the Mg‐HA motors efficiently alleviated joint damage, suppressed the overall severity of arthritis, and accelerated recovery, which might be related to the anti‐inflammatory mechanism of H_2_ inhibiting the transcription activity of NF‐κB. Nevertheless, it was not perfect for local H_2_ production because larger bubbles could potentially obstruct microvasculature.

In addition, this H_2_ treatment strategy can also be used to protect cells from inflammatory damage. For example, Jia et al. constructed AB‐loaded hollow carbon‐based nanoparticles (HC‐AB NPs) hydrogenothermal synergistic therapy guided by NIR‐II photoacoustic (PA) imaging.^[^ ^138^ ^]^ The HC‐AB NPs possessed good NIR‐II absorption capabilities, which benefited spatiotemporal resolution and tissue penetration depth, thus enhancing therapeutic efficacy. After administration, the acidic‐sensitive HC‐AB NPs released H_2_ continuously. Meanwhile, the heat generated by NIR‐II laser irradiation accelerated this process. The produced H_2_ was used for gas therapy, which significantly decreased the levels of pro‐inflammatory cytokines (TNF‐α, IL‐1β, and IL‐6) in tumor cells, thereby attenuating PTT‐induced inflammatory damage. Thus, the HC‐AB NPs provided a new basis for highly effective NIR‐II hydrogenothermal therapy. However, the generation amount of H_2_ from this system was limited by the sacrificial agent loading capacity of hollow carbon‐based nanoparticles.

#### 4.2.2 Inhibition of ATP

**Figure 14:**
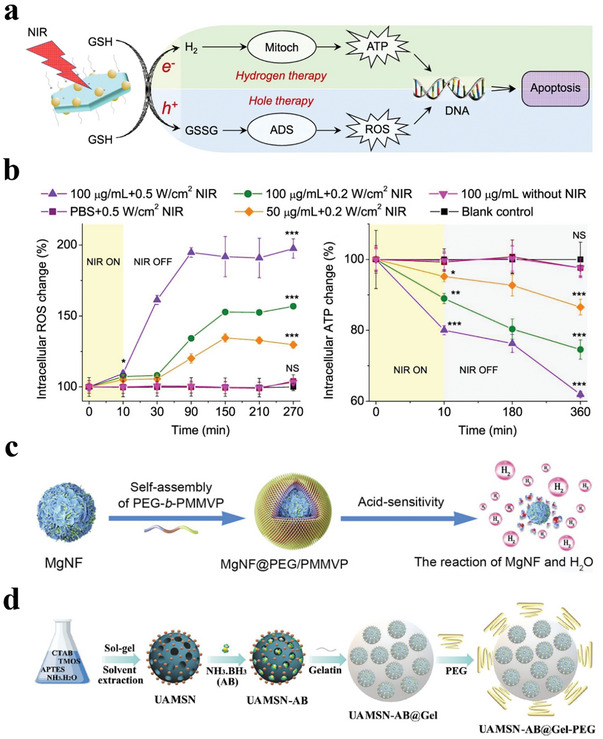
a) Combined hole/H_2_ therapy mechanisms of the SnS_1.68_‐WO_2.41_ nanocatalyst. b) The intracellular ROS and ATP levels after being treated with SnS_1.68_‐WO_2.41_ nanocatalyst. Reproduced with permission.^[^ ^110^ ^]^ Copyright 2021, Nature Publishing Group. c) Schematic illustration of the synthetic process of MgNF@PEG/PMMVP. Reproduced with permission.^[^ ^142^ ^]^ Copyright 2022, Elsevier. d) Schematic illustration of the preparation of the size‐switchable nanosystem (UAMSNAB@Gel‐PEG) for deep tumor therapy. Reproduced with permission.^[^ ^143^ ^]^ Copyright 2023, American Chemical Society.

In the tumor microenvironment (TME), ATP as a key substance for regulating tumor progression has become an emerging target for tumor treatments.^[^ ^139^ ^]^ ATP provides energy for the survival and proliferation of tumor cells and promotes tumor migration and metastasis.^[^ ^140^, ^141^ ^]^ Thus, ATP depletion has been considered a promising method to inhibit tumor growth. Interestingly, studies have proved that massive H_2_ could effectively inhibit the mitochondrial respiratory chain and reduce the generation of ATP of tumor cells, thus blocking cell energy and inducing apoptosis. According to this, biomaterial‐based H_2_ therapy is designed to decrease ATP selectively for cancer treatment. For example, the previously mentioned Z‐scheme SnS_1.68_‐WO_2.41_ nanocatalyst could inhibit ATP by NIR‐responsive released H_2_ for tumor treatment (**Figure** **14**a).^[^ ^110^ ^]^ Specifically, in vitro experiments revealed that the produced H_2_ gas caused severe damage to the mitochondria of 4T1 cells and reduced the intracellular ATP levels, thereby leading to the apoptosis of tumor cells (Figure 14b). After intravenous injection into the tumor‐bearing mice, the nanocatalyst significantly decreased intra‐tumoral ATP levels, further confirming the results of cell experiments. The stained experiments of tumor tissue slices showed that most of the tumor cells treated with SnS_1.68_‐WO_2.41_ plus NIR irradiation died without nuclei, suggesting that the released H_2_ could effectively kill tumor cells by depressing cell energy metabolism and increasing DNA damage. However, a therapeutically relevant amount of H_2_ was only produced with excessive amounts of nanomaterials. This requirement prevented further applications, especially against deep‐seated tumors, as well as targeted release.

Considering metallic Mg as a promising platform for H_2_ generation, Song et al. constructed polyethylene glycol‐b‐(poly(methyl methacrylate)‐co‐poly(4‐vinylpyridine) (PEG‐b‐PMMVP) coated Mg nanoflowers (MgNF@PEG/PMMVP) for H_2_ cancer therapy (Figure 14c).^[^ ^142^ ^]^ Under the acidic tumor environment, the PEG‐b‐PMMVP shell broke driven by the change of PMMVP from hydrophobic to hydrophilic, further exposing Mg nanoflowers, which reacted with water to produce large amounts of H_2_ gas. The experimental results proved that the released H_2_ could inhibit ATP generation by impairing mitochondrion, thus causing DNA damage and cell apoptosis. Moreover, the H_2_ bubbles improved ultrasound imaging signals, which was beneficial for imaging‐guided cancer therapy. However, this H_2_ generation process occurred on the interface, namely, the reaction began once reactants contacted each other, requiring encapsulated materials with good controllability and high stability.

A better anti‐tumor effect can be achieved through structural and functional design. Zhou et al. proposed a size‐switchable nanosystem (UAMSNAB@Gel‐PEG) to deliver H_2_ for deep tumor therapy (Figure 14d).^[^ ^143^ ^]^ This nanoplatform was constructed by AB‐loaded ultrasmall amino‐modified mesoporous silica nanoparticles (UAMSNAB) within a large gelatin (Gel) nanoparticle followed by covalent conjugation with PEG. Under the acidic condition of TME, AB could generate H_2_ gas for tumor tissue diffusion to realize effective hydrogen therapy. The in vitro and in vivo results confirmed that the released H_2_ from this nanosystem could induce mitochondrial damage and inhibit ATP production, thus enhancing tumor cell apoptosis and delaying tumor progression. This study provided a possible strategy of H_2_ therapy for deep tumor penetration. However, the therapeutic efficacy of H_2_ from these nondegradable vehicles was limited by their biocompatibility, loading efficiency, and tissue penetration capability.

#### 4.2.3 Inhibition of HSP

HSP is a molecular chaperone involved in the key mechanisms and progression of many tumors.^[^ ^144^ ^]^ Moreover, HSP could increase the drug resistance, heat resistance, and antioxidant capability of tumor cells to improve tumor survival.^[^ ^145^ ^]^ It has been reported that the activation of HSPs is dependent on ATP binding and hydrolysis at the N‐terminal domain of the protein.^[^ ^146^ ^]^ Thus, H_2_ could inhibit HSPs by destroying the mitochondrial respiratory chain and reducing ATP production for tumor treatment.

**Figure 15:**
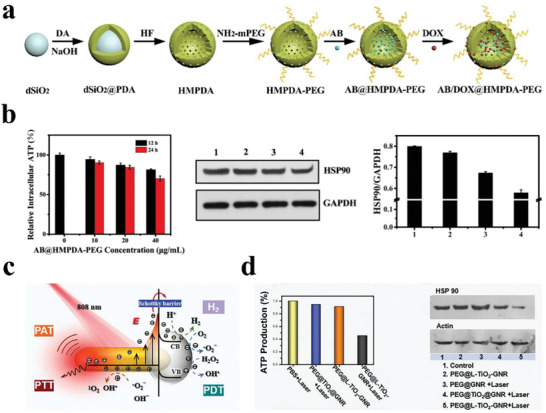
a) Diagram showing the synthesis of AB/DOX@HMPDA‐PEG. b) Therapeutic effect of AB/DOX@HMPDA‐PEG. Intracellular ATP levels after being treated with AB/DOX@HMPDA‐PEG. Western Blot analysis of intracellular HPS90 levels after different treatments. Reproduced with permission.^[^ ^147^ ^]^ Copyright 2021, Elsevier. c) Schematic illustration of the H_2_ generaion mechanism of L‐TiO_2_‐GNR nanoparticles. d) The inhibition rate of intracellular ATP after different treatments of L‐TiO_2_‐GNR nanoparticles. The levels of intracellular HSP90 after different treatments. Reproduced with permission.^[^ ^148^ ^]^ Copyright 2021, Wiley‐VCH.

For example, Yan et al. prepared acid‐responsive therapeutic nanoparticles (AB/DOX@HMPDA‐PEG) by co‐loading hollow mesoporous polydopamine (HMPDA) nanoparticles with PEG, doxorubicin (DOX) and AB for tumor therapy (**Figure** **15**a).^[^ ^147^ ^]^ On the one hand, AB/DOX@HMPDA‐PEG released H_2_ to act as a contrast agent for ultrasound imaging of the acidic tumor environment and tumor cell lysosomes. On the other hand, the massive release of H_2_ in lysosomes triggered the escape of the nanoparticles from the lysosomes, accompanied by the release of DOX, and blockaded the mitochondrial respiratory chain, which reduced the generation of ATP. Furthermore, the decreased ATP inhibited the expression of heat shock protein 90 (HSP90) to overcome the tumor cells' DOX resistance (Figure 15b). Experimental results proved that these nanoparticles realized ultrasound imaging of drug‐resistant tumors and controllable H_2_‐aided chemotherapy. Furthermore, this nanosystem to produce H_2_ was dependent on the acidity of the cell microenvironment that cancer cells usually possess, which restricted its application in other diseases.

In addition to overcoming drug resistance, inhibiting HSP production through H_2_ can also reduce the heat resistance of tumor cells. As an example, Ge et al. designed a gold nanorod/titanium dioxide (L‐TiO_2_‐GNR) nanosystem for the treatment of hypoxic tumors (Figure 15c).^[^ ^148^ ^]^ Specifically, the TiO_2_ was controlled by single‐head growth on gold nanorods to form the Schottky junction. Under the 808 nm laser irradiation, the generated hot electrons from gold nanorods overcame the Schottky barrier to transfer into the CB of TiO_2_ for H_2_ production. The released H_2_ blocked the synthesis of ATP by damaging the mitochondria, which could effectively decrease HSP90 and inhibit HSP90 activation, thus overcoming the heat resistance of tumor cells (Figure 15d). The in vivo experiments showed that this nanosystem had enhanced PDT and PTT effects for tumors with the help of H_2_ therapy, especially for hypoxic solid tumors.

In conclusion, different triggers such as pH and light have been performed for controlled and sustained release of H_2_ in the inhibition strategies. In addition to its inhibition effects, H_2_ can be combined with other modalities to enhance therapeutic efficacy and reduce side effects.

### 4.3 Regulation Strategies

**Figure 16:**
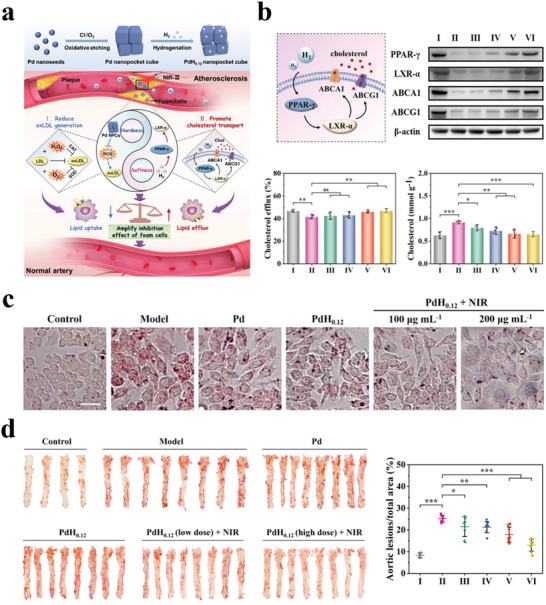
a) Strategy of “coupling hardness and softness” of foam cell amplified inhibition using PdH_0.12_ NPCs to attenuate atherosclerosis. b) In vitro therapeutic effect of PdH_0.12_ NPCs. Schematic illustration of NIR‐responsive H_2_ released from PdH_0.12_ NPCs to regulate the cholesterol transport pathway. Western blotting analysis of PPAR‐γ, LXR‐α, ABCA1, and ABCG1 protein levels in RAW264.7 cells after different treatments. Intracellular cholesterol efflux and total cholesterol levels after different treatments. c) Optical microscopy images of oxLDL‐induced foam cell formation after different treatments. d) In vivo therapeutic effect of PdH_0.12_ NPCs. Representative photographs of ORO‐stained en face aortas from mice after different treatments. Quantitative analysis of the lesion area in aortas. Reproduced with permission.^[^ ^149^ ^]^ Copyright 2021, Wiley‐VCH.

As a gaseous signaling molecule, H_2_ can regulate cellular signaling pathways and expressions of some factors, such as the NF‐κB pathway, the Nrf2/HO‐1 pathway, and the expression of miRNA, etc. Encouraged by this, biomaterial‐based H_2_ therapy has recently been developed as regulatory molecules to treat diseases. In our recent work, we reported Pd hydride nanopocket cubes (PdH_0.12_ NPCs) to activate the lipid metabolic pathway for atherosclerosis treatment (**Figure** **16**a).^[^ ^149^ ^]^ As a chronic disease, the primary manifestation of atherosclerosis is the accumulation of lipids in the artery wall, which induces systemic autoimmunity, causing long‐term chronic inflammation.^[^ ^150^ ^]^ Therefore, we used a “coupling hardness with softness” strategy by applying PdH_0.12_ NPCs to affect the uptake and efflux of lipids. The unique shape of the PdH_0.12_ NPCs regulated the NIR absorption and increased the antioxidant enzyme activities of the carrier. The PdH_0.12_ NPCs exerted the “hardness” role by scavenging ROS based on their antioxidant enzyme activities. Under irradiation by a NIR laser, the increase in the local temperature abrogated the Pd‐H binding force, triggering H_2_ release. The H_2_ produced from PdH_0.12_ NPCs upregulated the peroxisome proliferator‐activated receptor gamma (PPAR‐γ)‐mediated cholesterol transport pathway, thereby exerting the “softness” function (Figure 16b). Taken together, ROS scavenging decreased lipid uptake and enhanced cholesterol transport increased the efflux of lipids, ultimately suppressing foam cell formation (Figure 16c). In vitro and in vivo experimental results indicated that PdH_0.12_ NPCs treated with NIR irradiation could notably relieve the progression of atherosclerotic lesions (Figure 16d).

**Figure 17:**
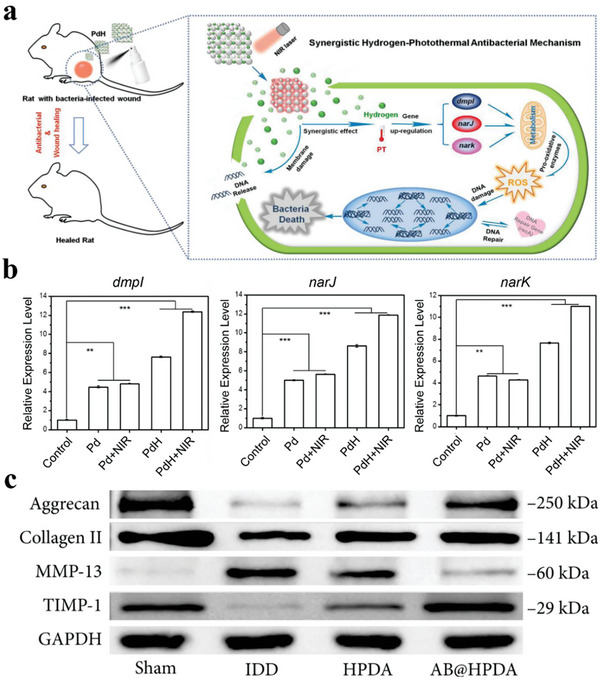
a) Diagram showing the application of PdH nanohydride for antibacterial and wound‐healing in rats with the bacteria‐infected wound. b) Therapeutic effect of PdH nanohydride. The relative ROS levels and *dmpI*, *narJ*, and *nark* expression in *S. aureus* after different treatments. Reproduced with permission.^[^ ^151^ ^]^ Copyright 2019, Wiley‐VCH. c) Western blotting analysis of collagen II, aggrecan, MMP‐13, and TIMP‐1 protein expression. Reproduced with permission.^[^ ^152^ ^]^ Copyright 2023, Hindawi.

Moreover, active H_2_ has been demonstrated to be highly efficient in antibacterial and wound‐healing applications by regulating the expressions of bacterial metabolism‐relevant genes. Xue et al. constructed Pd nanocubes and incorporated H_2_ into their lattice to fabricate PdH nanohydride for synergistic H_2_‐photothermal antibacterial and wound‐healing therapies (**Figure** **17**a).^[^ ^151^ ^]^ The synthesized PdH exhibited an NIR laser‐controlled active H_2_ gas release capability, in which the H_2_ release rate can be tuned on demand via the NIR laser power density and irradiation time. An in‐depth study revealed the potential antibacterial mechanism of synergistic H_2_‐photothermal effect. The PdH could activate metabolism‐associated genes, such as *dmpI*, *narJ*, and *nark* in the bacteria, which encode oxidative metabolic enzymes that produce increased ROS to induce DNA damage (Figure 17b). More importantly, the combined H_2_‐photothermal effect resulted in severe damage to the bacterial membrane, which promoted H_2_ diffusion and intracellular compound release. This demonstrated that the obtained PdH had marked therapeutic efficacy in healing wounds of rats with a severe bacterial infection.

Additionally, H_2_ is effective for other specific disease treatments. Wang et al. developed an ammonia borane‐loaded hollow polydopamine (AB@HPDA) for intervertebral disc degeneration (IDD) treatment by producing H_2_ to regulate the expression of associated proteins.^[^ ^152^ ^]^ As previously mentioned, AB could release H_2_ in an acid‐responsive pattern. After injection, the produced H_2_ from AB@HPDA could significantly block the disc extracellular matrix (ECM) degradation by upregulating the expression of anabolic proteins (such as collagen II, aggrecan, and tissue inhibitor of metalloproteinase‐1 (TIMP‐1)) and downregulating the breakdown level of metabolic proteins such as matrix metalloproteinase‐13 (MMP‐13) for IDD treatment (Figure 17c). This study provided a potential direction for H_2_ therapy. As a result, these studies further demonstrated the signal transduction role of H_2_ molecules in disease treatment.

## 5 Conclusion and Perspectives

H_2_ therapy is rapidly developing, and advanced methods of H_2_ delivery will have a crucial function in improving H_2_ therapy availability and efficacy. The use of biotechnology in H_2_ therapy is emerging as a promising therapeutic strategy. Compared with traditional H_2_ administration methods, biomaterial‐mediated H_2_ therapy has certain advantages, including better pharmacokinetic parameters and tissue specificity, more effective H_2_ concentration in solution, higher H_2_ delivery efficiency, and increased accessibility of the implementation. Also, their therapeutic effectiveness has been demonstrated according to different types of disease models. This review details the important recent advances in the emerging field of stimuli‐responsive H_2_‐releasing biomaterials, including spontaneous release, endogenous stimuli (for example, pH and water), and exogenous stimuli (for example, light, NIR, and US). These controlled H_2_ release techniques allow for on‐demand gas therapy and H_2_‐sensitized synergistic therapy. In addition, an emphasis on the mechanisms of biomaterial‐based H_2_ therapy has been discussed in this review, which covers free radicals scavenging strategy (for example, ROS and LPO), danger factors inhibition strategy (for example, pro‐inflammatory cytokines, ATP, and HSP), and signal molecules regulation strategy (for example, cholesterol transport pathway, metabolism‐relevant genes, and disease‐associated proteins). Although biomaterial‐based H_2_ therapy has exhibited good performance, it is still in the fundamental and exploratory stage with many challenges and opportunities, which will encourage future research and development in the following five directions:

Biomedicines with a stable biological response and a precise H_2_ therapeutic effect should be further studied. Several biomaterials are suitable as H_2_ generators and efficiently store H_2_; however, improved biocompatibility and in vivo stability are required for their therapeutic application. For instance, introducing biomaterials might increase drug delivery and release diffusion complexity; however, their effects on cells, tissues, and organs during transport in organisms remain unclear. In addition, the original effect of biomaterials should be carefully considered before further clinical translation, which might affect the initial therapeutic mechanism of H_2_. Furthermore, considering the complexity of biological systems and the metabolism of biomaterials, more attention should be paid to the development of biomaterial‐free H_2_ generation and delivery systems to reduce their uncertainty in vivo. At present, using electrochemical technology to locally release high‐purity H_2_ is one of the major biomaterial‐free strategies, which have achieved ideal results in tumor treatment.^[^ ^42^, ^153^ ^]^ However, currently the method may not be valid for the treatment of non‐solid tumors such as lung cancer and liver cancer, and other diseases. Therefore, how to achieve high concentration and good biosafety of H_2_ delivery needs to be further investigated, especially how to design an efficient H_2_ production and targeting strategy to disease tissues for H_2_ therapy is an urgent problem to be solved.

The control of H_2_ delivery and release should be enhanced. The aim of introducing biotechnology and biomaterials is to realize controllable H_2_ delivery, release, and therapy. Currently, bioreactor‐based H_2_ production can occur via 1) self‐catalysis, 2) chemical reactions such as Mg reacting with water and Zn reacting with acid, and 3) photocatalysis, 4) ultrasonic catalysis. However, these strategies are insufficient for clinical treatments. Studies on the control of H_2_ release through the stimuli‐response need to be further developed, especially for targeted H_2_ delivery. For example, photo‐triggered H_2_ therapy is only efficient for superficial diseases due to the limited tissue penetration capacity of light. The sensitivity and controllability of physical microenvironment‐responsive H_2_ therapy need to be further improved due to the obvious difference between various diseases at different stages. Therefore, it is necessary to design safe and effective therapeutic strategies by optimizing the stability of biomaterials, improving their H_2_ storage capacity, and controlling intelligent H_2_ release. More exogenous and endogenous triggers for H_2_ therapy should be investigated in further research.

The therapeutic process and mechanism of H_2_ still need to be further explored. As an emerging therapeutic modality, H_2_ therapy is still at the basic research stage, but it is worth further promoting its clinical translation due to its high efficacy and biosafety. The primary mechanism of H_2_ therapy is now widely accepted to rely on selective antioxidant and anti‐inflammatory effects. Compared with other antioxidants, such as vitamins, the advantage of H_2_ is that it can selectively remove highly toxic ·OH and ONOO─ that lead to oxidative damage without affecting ROS. However, as a signaling molecule, some as‐yet‐undefined biological mechanisms of H_2_ remain to be explored and revealed, such as the connection between its anti‐inflammatory and antioxidant properties, targets, mode of action, and the associated in vivo metabolic pathways. Furthermore, the effectiveness of biomaterial‐assisted H_2_ therapy is based on the whole therapeutic effect rather than the corresponding detailed molecular, physical, and biological mechanisms, which greatly impedes the further optimization of H_2_ therapy performance. Therefore, the discovered mechanisms of H_2_ are insufficient to elucidate its efficacy, which requires more fundamental studies.

The synergistic effects of combining H_2_ therapy with other treatments should be the focus of future research to maximize performance. Recently, certain traditional therapeutic modalities have been combined with H_2_ therapy to augment the treatment of diseases, such as PTT, PDT, and chemotherapy. Combination with other new biomedical therapies still needs to be investigated. For instance, H_2_ can sensitize drug‐resistant cells that have and may lead to the induction of an immunological response against distal tumors. Thus, the combination of immunotherapy and H_2_ therapy should be explored as a promising strategy. In addition, there is an urgent need to employ various biomedical technologies to monitor and detection of H_2_ in real‐time for precise disease diagnosis and treatment based on multifunctional H_2_‐generating platforms.

The biosafety of biomaterials should be carefully evaluated to promote their clinical translation. At present, only a very few therapeutic biomaterials have been applied in clinical trials. Although H_2_ gas has been proven to be safe and biocompatible, biomaterials may have biosafety concerns. For instance, the most commonly used H_2_‐generating platforms are inorganic materials. Although relatively stable, they may be potentially toxic to normal cells, tissues, or organisms due to the presence of metal elements. The initial and long‐term biocompatibility and biosafety of these biomaterials should be explored. Besides sufficient data on these aspects need to be provided before their clinical translation and application. Moreover, the relationship between the biological effects, biosafety, biodistribution, and metabolism of H_2_‐releasing biomaterials should be systematically and comprehensively elucidated. Therefore, more efforts should be put into biosafety and biocompatibility in the following design, construction, and performance evaluation of H_2_‐releasing biomaterials.

More research is necessary to reveal the complex mechanisms of interaction between hydrogen molecules, diseased cells, and healthy cells in the future. It seems that severe diseases can indeed be treated well with H_2_ therapy, but healthy people can feel a minimum change after H_2_ administration, indicating that the hydrogen molecule is a kind of homeostatic regulator. Hence, there is an urgent need to understand why hydrogen molecule exhibits such selectivity in many disease models. This knowledge will help design safe and effective biomaterial‐based H_2_ therapy targeting specific diseases. Moreover, most studies evaluating the efficacy of biomaterial‐based H_2_ therapy have been conducted in animal disease models, which should be carefully considered to improve the transition from preclinical research to the clinic.

In conclusion, although there are a few unresolved barriers, H_2_ therapy is still an emerging research frontier that deserves further exploration. In particular, biomaterial‐based H_2_ delivery strategies provide a great possibility to realize precise and controlled release of H_2_ at specific sites. It can be predictable that with continuous efforts, biomaterial‐based H_2_ therapy systems are likely to become a useful clinical therapeutic tool for disease prevention and treatment.

## Conflict of Interest

The authors declare no conflict of interest
